# Comprehensive analysis of oxidative stress-related lncRNA signatures in glioma reveals the discrepancy of prognostic and immune infiltration

**DOI:** 10.1038/s41598-023-34909-y

**Published:** 2023-05-12

**Authors:** Zhenyi Shi, Yingying Wu, Qingchan Zhuo, Yufang Zuo, Jiong Lin, Huadi Shi, Hechao Zhou, Zumin Xu

**Affiliations:** Cancer Center, Affiliated Hospital of Guangdong Medical University, Zhanjiang, 524000 Guangdong People’s Republic of China

**Keywords:** Cancer, Computational biology and bioinformatics, Immunology

## Abstract

Oxidative stress refers to the process of reactive oxide species (ROS) increase in human body due to various factors, which leads to oxidative damage in human tissues. Current studies have confirmed that sustained oxidative stress is one of the distinctive features throughout the development of tumors. Numerous reports have shown that lncRNAs can regulate the process of oxidative stress through multiple pathways. However, the relationship between glioma-associated oxidative stress and lncRNAs is not clearly investigated. RNA sequencing data of GBM (glioblastoma) and LGG (low grade glioma) and corresponding clinical data were retrieved from the TCGA database. Oxidative stress related lncRNAs (ORLs) were identified by Pearson correlation analysis. Prognostic models for 6-ORLs were structured in the training cohort by univariate Cox regression analysis, multivariate Cox regression analysis and LASSO regression analysis. We constructed the nomogram and verified its predictive efficacy by Calibration curves and DCA decision curves. The biological functions and pathways of 6-ORLs-related mRNAs were inferred by Gene Set Enrichment Analysis. Immune cell abundance and immune function associated with risk score (RS) were estimated by ssGSEA, CIBERSORT and MCPcounter synthetically. External validation of the signature was completed using the CGGA-325 and CGGA-693 datasets. 6-ORLs signature—AC083864.2, AC107294.1, AL035446.1, CRNDE, LINC02600, and SNAI3-AS1—were identified through our analysis as being predictive of glioma prognosis. Kaplan–Meier and ROC curves indicated that the signature has a dependable predictive efficacy in the TCGA training cohort, validation cohort and CGGA-325/CGGA-693 test cohort. The 6-ORLs signature were verified to be independent prognostic predictors by multivariate cox regression and stratified survival analysis. Nomogram built with risk scores had strong predictive efficacy for patients' overall survival (OS). The outcomes of the functional enrichment analysis revealing potential molecular regulatory mechanisms for the 6-ORLs. Patients in the high-risk subgroup presented a significant immune microenvironment of macrophage M0 and cancer-associated fibroblast infiltration which was associated with a poorer prognosis. Finally, the expression levels of 6-ORLs in U87/U251/T98/U138 and HA1800 cell lines were verified by RT-qPCR. The nomogram in this study has been made available as a web version for clinicians. This 6-ORLs risk signature has the capabilities to predict the prognosis of glioma patients, assist in evaluating immune infiltration, and assess the efficacy of various anti-tumor systemic therapy regimens.

## Introduction

Glioma is the primary cancer of the central nervous system (CNS) and is the most common brain tumor in adults (6/100,000). According to the World Health Organization (WHO) 2016 classification, gliomas can be divided into diffuse gliomas and non-diffuse gliomas, and diffuse gliomas are further divided into low-grade gliomas (LGG) and glioblastomas (GBM)^1^. Of these, GBM, the most common and most lethal (3/100,000), is known for being extremely aggressive with a very poor prognosis and a median survival of only about 1 year^2,3^. Currently, the accepted standard treatment paradigm for GBM consists of gross total tumor resection, postoperative chemotherapy with temozolomide (TMZ) and concurrent radiation therapy, with subsequent TMZ maintenance chemotherapy. Even though important molecular features have been used for molecular typing of gliomas and development of treatment, the standard treatment paradigm and prognosis of glioma remains unchanged significantly^4^.Targeted therapies and immunotherapy, despite being considered to have potential prognostic value, have not yet been identified to significantly improve prognosis^4,5^. Therefore, there is an urgent need to find new molecular targets or prognostic markers for the diagnosis and treatment of glioma.

Oxidative stress (OS) is a state in which the level of reactive oxygen species (ROS) in cells is superfluous and exceeds their own antioxidant defense capacity^6^. Oxidative stress can activate inflammation-related signaling pathways and is also closely associated with tumorigenesis and development. Oxidative stress has been reported to activate the expression of multiple transcription factors and activate inflammation-related pathways, leading to enhanced tumor cell proliferation, invasion, chemoresistance and radioresistance^7^. It has been noted that, ROS produced by macrophages are involved in all stages of the carcinogenic process^8,9^. Therefore, it is reasonable to assume that the oxidative stress process may have a regulatory role in tumorigenesis and progression, and its related regulatory genes may become molecular targets for cancer therapy.

Long-stranded non-coding RNA (lncRNA) is a collective term for a set of transcripts of non-coding proteins, with transcripts longer than 200 nucleotides^10^. In recent years, lncRNAs have been shown to play important regulatory roles in biological functions such as transcription, translation, RNA metabolism, epigenetics, embryonic development, stem cell maintenance and differentiation, apoptosis and cellular autophagy^11^, and have been closely associated with cancer metabolism, cardiovascular disease, neuropathy and many other disease processes^12–14^. Toll-like receptors (TLRs) are a class of protein molecules that play a key role in innate immunity, and it has been shown that there are lncRNAs regulated by TLR-related genes that mediate innate immune function and inflammatory responses^15,16^. The above studies suggest that there are likely to be undiscovered regulatory targets in lncRNAs related to inflammatory response, oxidative stress, and cancer progression, which is an area worth exploring.

Glioma Stem Cells (GSC) are a specific type of tumor cells that have the strongest differentiation potential in glioma, have a high self-renewal capacity, and can differentiate into a variety of heterogeneous glioma cells, bringing great variability to the tumor heterogeneity and tumor microenvironment in glioma. In contrast to cells without stem cell characteristics, GSC may exhibit greater intrinsic tolerance to therapeutic treatment due to inherent properties and adaptive resistance pathways, thereby promoting tumor progression, invasion and recurrence^17^. The various cellular components of the microenvironment, such as neuronal precursor cells, microglial cells, macrophages, fibroblasts, and multiple immune cells such as dendritic cells, leukocytes, or natural killer cells, together form the tumor microenvironment (TME) of glioma, which synergistically promotes tumor growth, proliferation, and therapeutic resistance^18,19^. Among the TME of glioma, hypoxia has a considerable importance. It has been noted that, especially in high-grade glioma, the upregulation of hypoxia-inducing factor (HIF) often predicts the emergence of a more aggressive and drug-resistant phenotype^20^. In a cellular hypoxic environment, mitochondria open oxidative phosphorylation-related channels and become the main energy supply to ensure cell survival, and blocking the oxygen supply pathway of the TME in glioma by inhibiting this pathway is expected to suppress tumor aggressiveness and improve radiotherapy resistance^21^. The hypoxic characteristics of glioma result in a substantial increase in the activity of multiple pro-carcinogenic pathways in their TME. GSC, as well as other tumor stem cells, are thought to be more adapted to hypoxic, lactate-rich TME and promote tumor proliferative activity^22^. The core regulators of the hypoxic phenotype of glioma, HIF 1-α and HIF-2 α, regulate angiogenesis and resistance to the acidic environment mainly in TME, upregulating VEGF, IL-8, and the stem cell marker of tumors, CD133^23,24^. In conclusion, hypoxia and oxidative stress in glioma are likely to promote its highly malignant phenotype and act synergistically with TME to further increase tumor aggressiveness and treatment resistance.

In this study, we obtained a predictive signature of oxidative stress-related lncRNAs in glioma, demonstrated the ability to predict the prognosis of glioma patients, preliminarily explored its relationship with tumor immunity and immunotherapy, indicating its potential to further guide clinical diagnosis and treatment strategies.

## Materials and methods

### Patient datasets and clinical data

We downloaded the Transcripts Per Million (TPM)-standardized RNA-seq data and the corresponding clinical and prognostic data for The Cancer Genome Atlas glioblastoma and low-grade glioma (TCGA-GBM and TCGA-LGG) dataset from the TCGA website (https://portal.gdc.cancer.gov/), downloaded the STAR read counts RNA-seq data and the corresponding clinical and prognostic data for the Chinese Glioma Genome Atlas mRNAseq_325^25–27^ and mRNAseq_693^27–29^ (CGGA-325 and CGGA-693) dataset from the CGGA website (http://www.cgga.org.cn/). Subsequently, based on the non-redundant exon length provided by the GENCODE V19 human genome annotation data from GENCODE website (https://www.gencodegenes.org/), the STAR read counts RNA-seq data from CGGA were used to calculate the TPM according to the following formula:

$$TPMi=\frac{\mathrm{Ni}/\mathrm{Li}\times {10}^{6}}{\mathrm{sum}(\mathrm{N}1/\mathrm{L}1+\mathrm{N}2/\mathrm{L}2+\dots +\mathrm{Nn}/\mathrm{Ln})}$$

where TPM is the TPM value of a transcript in a sample; N represents the number of reads of the transcript; L denotes the non-redundant exon length corresponding to the transcript; and n refers to the number of all transcripts in the sample. The CGGA RNA-seq dataset in this study was analyzed with the transformed TPM values. To reduce statistical bias in this analysis, all patients with missing overall survival (OS) values in the TCGA and CGGA cohorts were excluded. In addition, samples lacking other clinical characteristics will be excluded from the stratified analysis. In the TCGA, CGGA-325 and CGGA-693 cohorts, there were 661, 313 and 657 samples for survival analysis as well as 564, 286 and 429 samples for stratified analysis of clinical characteristics, respectively. Subsequently, all patients in TCGA were randomly divided into a training cohort (332 samples) and a validation cohort (329 samples) for regression analysis and construction of prognostic signatures using the "caret" package in R (version 4.1.0, https://www.r-project.org/). 825 oxidative stress-related genes were downloaded from the GeneCards database (https://www.genecards.org/), and those with a relevance score greater than 7 were selected.

### Identification of differentially expressed oxidative stress-related genes

We intersected 825 oxidative stress-related genes with RNA-seq data from the TCGA cohort and analyzed the intersected genes using the "limma" package in R, with false discovery rate (FDR) < 0.05 and |log2 fold change (FC)| > 1 as screening criteria to obtain oxidative stress-related differentially expressed genes (DEGs).

### Functional enrichment analysis of DEGs

We used the "clusterProfiler" and "org.Hs.e.g.db" packages in R to complete functional enrichment analysis of DEGs for Gene Ontology (GO) and Kyoto Encyclopedia of Genes and Genomes (KEGG)^30–32^ and visualized the results using the "enrichplot" and "ggplot2" packages in R.

### Construction of the Necroptosis-Related lncRNA Predictive Signature

We used the "limma" package in R to calculate the Pearson correlation between lncRNAs and DEGs in the TCGA cohort and obtained 3073 oxidative stress-related lncRNAs (ORLs) using Pearson correlation coefficient > 0.3 as a screening condition. The univariate Cox regression analysis was performed with the "survival" package in R, and 1447 prognosis-related ORLs were identified in the TCGA cohort using chi-square and log-rank tests with P value < 0.001 as screening criteria. Using the "glmnet" package in R, the LASSO algorithm was used to dimensionality reduce the prognosis-related ORLs to remove overfitting genes, resulting in 64 ORLs. For the samples in the TCGA training cohort, multivariate Cox regression analysis was performed on 64 ORLs, with log-rank P value < 0.05 as the screening criterion, resulting in a final panel of 6 ORLs. Subsequently, the risk scores (RS) of the 6-ORLs were calculated to construct a Cox proportional hazards regression model. The formula for this analysis is as follows:

$$\mathrm{Risk\,\, score }= \sum_{i=1}^{n}(Coefi\times xi)$$

Coef represents the value of the coefficient associated with risk, x represents the expression value of the selected ORL. This formula was used to calculate the RS for each glioma patient. survival analysis was performed on samples from the training and validation cohorts using the "survival" package in R to classify patients into high- and low-risk groups based on the median RS, and Kaplan–Meier (K–M) curves were plotted using the "survminer" package in R. Using the "timeROC" package in R, the area under the ROC curve (AUC) was calculated to assess the sensitivity and specificity of 6-ORLs characteristics for survival prediction of patient prognosis. The 6-ORLs signature was verified in the CGGA-325 and CGGA-693 cohorts with the same analysis method. The heatmap of 6-ORLs expression levels and distribution of RS and clinicopathological variables was performed by the "ggplot2" package in R.

### Nomogram construction and validation

To verify the independence of the predictive signature of 6-ORLs, we combined RS with clinicopathological characteristics such as age, gender, WHO classification, IDH mutation, 1p 19q co-deletion and MGMT promoter methylation to construct a nomogram using the "rms" package in R to predict 1-, 3- and 5-year survival in glioma patients, and calibration curves were drawn to compare nomogram-predicted 1-, 3-, and 5-year survival probabilities with the actual observed outcomes. In addition, we used the "ggDCA" package in R to plot DCA decision curves to assess the validity of nomogram for treatment decisions. Subsequently, the nomogram was created as a web version using the "DynNom" package in R and uploaded to (https://dnszy.shinyapps.io/Glioma_OS_lncRNA/) for online use.

### Gene set enrichment analysis

Using the "limma" package in R, differential expression analysis was completed for the high-risk group (333 patients) and the low-risk group (328 patients), with FDR < 0.05 as the screening criterion and no restriction on log2FC, resulting in 16,343 genes and corresponding log2FC values. Subsequently, the "org.Hs.eg.db" package, "clusterProfiler" package and "ReactomePA" package in R were used to complete the gene set enrichment analysis (GSEA). The enrichment scores (ES) of GO, KEGG and Reactome terms were obtained by using P value < 0.05 as the screening criteria. The top 10 terms of ES were visualized using the "gseaplot2" function in R.

### Principal component analysis

To demonstrate whether the expression level of total RNA, oxidative stress-associated mRNA and ORL differed significantly among the high/low risk groups, we performed principal component analysis (PCA) on all samples, used the "scatterplot3d" package in R to complete the analysis and visualization.

### Immune cell infiltration and immune function analysis

We used single sample gene set enrichment analysis (ssGSEA), CIBERSORT and Microenvironment Cell Populations-counter (MCP-counter) algorithm to comprehensively assess TCGA cohort and the CGGA cohort for differences in immune cell infiltration and immune function in the high/low risk group. As a nonparametric and unsupervised method, gene set variation analysis (GSVA) is primarily used to assess gene set enrichment score (ES) based on gene expression profiles from microarrays or RNA-seq data^33^. We used the "ssGSEA" method^34^ in the "GSVA" package in R to assess the infiltration fraction of 16 immune cells and the activity of 13 immune-related pathways by calculating the ES of each single sample. We used the CIBERSORT algorithm in R to estimate immune cell infiltration in all glioma samples. This method, first proposed by Newman et al., uses the cell-specific gene signatures to discriminate a total of 22 immune cell populations^35^. The 22 immune cell typologies of CIBERSORT were combined into four typologies of dendritic cell, lymphocytes, macrophage and mast cell according to the method provided by Bailiang Li et al.^36^. In addition, we assessed the abundance of various immune and stromal cell populations based on RNA-seq data for each sample through the "MCPcounter" package in R to derive relative infiltration of fibroblasts, endothelial cells, and eight additional immune cell populations^37^. We analyzed the differences in expression level of 42 immune checkpoint-associated genes in high/low risk groups to investigate possible correlations between immune checkpoints and RS. Different expression levels of immune checkpoint-associated genes may predict sensitivity to immune checkpoint inhibitors. The visualization of immune cell infiltration histogram, boxplot, correlation plot and heatmap were performed by the "ggplot2" package in R.

### RT-qPCR

Total RNA was extracted using TRIzol reagent (Invitrogen) and reverse transcription of RNA was completed using the PrimeScirpt Reverse Transcription Kit (TaKaRa Bio, Japan) according to the manufacturer's instructions. RT-qPCR was operated using Real-Time PCR reagent TB GreenTM Premix Ex TaqTM II kit (TaKaRa Bio, Japan). PCR reactions were performed using ABI 7500 Real-Time PCR system. Gene expression levels were calculated using the 2-ΔΔCT method. The primers of SNAI3-AS1 were referred to the study of Yarui Li et al. [SNAI3-AS1 primers], and the rest of the lncRNA primers were designed by the online tool of National Center for Biotechnology Information website (https://www.ncbi.nlm.nih.gov/). The following lncRNA primers were used for this experiment:

SNAI3-AS1 Forward: 5′-GCGTTATGTCGTTTGGTTGATG-3′.

SNAI3-AS1 Reverse: 5′-TGGCAGGAATGAGGTGAGC-3′.

AL035446.1 Forward: 5′-GAAAAATGCGTGCCCTCTTGT-3′.

AL035446.1 Reverse: 5′-TCTTGGGCTCCATCATTCATCC-3′.

AC107294.1 Forward: 5′-AAGCTCCCTCAAACAGGCCC-3′.

AC107294.1 Reverse: 5′-TGCAGTGTAACAGAGCTGCG-3′.

AC083864.2 Forward: 5′-GCATGAGGTGTTTCCTCCCA-3′.

AC083864.2 Reverse: 5′-CTGCAACCCAAGGCAATGAC-3′.

LINC02600 Forward: 5′-AGTACATGGCAGAAGTGGGC-3′.

LINC02600 Reverse: 5′-TGAGATACAGGCAGGTTCGC-3′.

CRNDE Forward: 5′-CCCACCCTGGAAACTCCTAC-3′.

CRNDE Reverse: 5′-GTCCCTAGAGAAGAGGCGAC-3′.

### Statistical analysis

We completed all analyses using the statistical software R (version 4.1.0). Welch t-test, Wilcoxon rank sum test, or chi-square test were used to verify the differences. Kaplan–Meier survival curves were assessed by using the log-rank test. Both univariate and multivariate Cox regression analyses were used to identify independent prognostic factors. We used the "survivalROC" package in R in order to create ROC curves and calculate AUC values to verify their predictive ability.

## Results

### Identification of ORLs in glioma and biological function analysis

**Figure 1:**
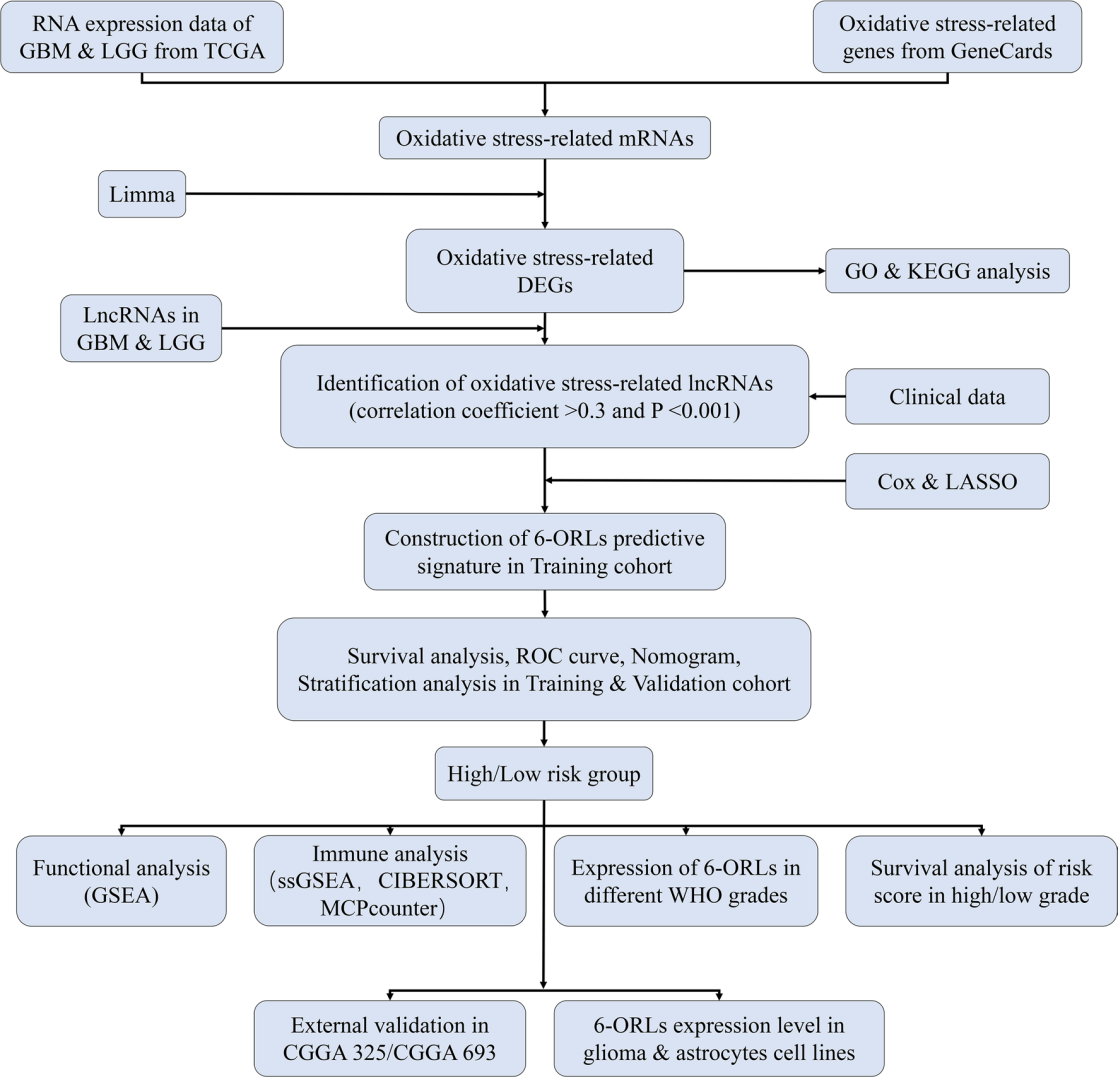
The flowchart of the research. *GBM* glioblastoma, *LGG* low grade glioma, *TCGA* The Cancer Genome Atlas, *DEGs* differentially expressed genes, *GO* Gene Ontology, *KEGG* Kyoto Encyclopedia of Genes and Genomes, *lncRNAs* long noncoding RNAs, *ORLs* oxidative stress-related lncRNAs, *ROC* receiver operating characteristic, *GSEA* gene set enrichment analysis, *CGGA* Chinese Glioma Genome Atlas.

**Figure 2:**
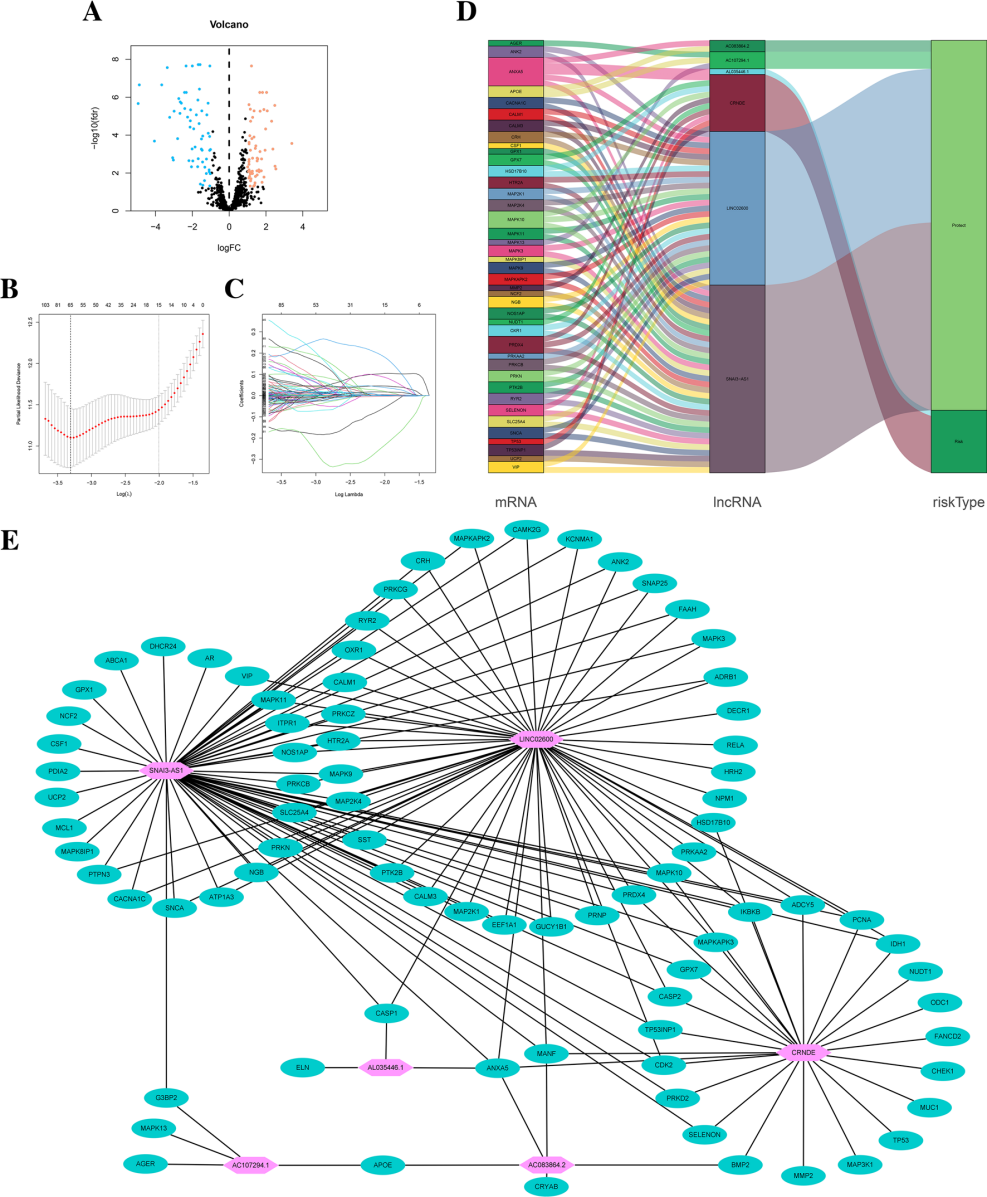
Volcano plot, LASSO regression analysis and gene association network of 6-ORLs. (**A**) Volcano plot of 120 OR-DEGs in TCGA glioma cohort. (**B**) The partial likelihood deviance with changing of log(λ). (**C**) LASSO coefficient profiles of oxidative stress-associated lncRNAs. (**D**) Sankey diagram of 6-ORLs, OR-DEGs and risk type. (**E**) Relationship network of 6-ORLs and OR-DEGs.

The flowchart for our study is shown in Fig. 1. We obtained 120 oxidative stress-related DEGs (OR-DEGs) in TCGA-GBM and TCGA-LGG RNA-seq data, including 60 upregulated genes and 60 downregulated genes (Fig. 2A). We performed GO and KEGG analyses of OR-DEGs. The enrichment ranking of each pathway was derived based on the P value and the number of enriched genes. We show the top 10 terms in the biological process (BP), cellular component (CC), and molecular function (MF) of GO, and the top 20 pathways in KEGG (Fig. S1A,B). Among them, the most enriched BP, CC and MF terms are "response to oxidative stress", "neuronal cell body" and "protein serine/threonine kinase activity" respectively, and the most enriched KEGG pathway is "GnRH signaling pathway", indicating that OR-DEGs may be highly associated with oxidative stress-related biological functions, neuronal activity, TGF-β signaling pathway and GnRH-related cellular activity. A total of 13,975 lncRNAs were identified in the TCGA cohort based on the GTF annotation file of human lncRNAs. 3,073 oxidative stress-related lncRNAs (ORLs) were subsequently identified by Pearson correlation analysis (Supplementary Table S1).

### Construction of the ORLs prognostic signature

We used univariate Cox regression analysis to identify 1447 ORLs that were strongly associated with prognosis in the TCGA cohort of glioma patients. Baseline characteristics of the patients are shown in Table 1. We show the crossvalidatederror of the LASSO regression model by coefficient profile plot (Fig. 2B). Subsequently, the most normalized and most brief model, with a crossvalidatederror within 1 standard error of the minimum are selected, as shown by the vertical dashed line in Fig. 2C. Then, overfitting genes were removed by using LASSO regression analysis to obtain 65 ORLs (Fig. 2B,C). Afterwards, multivariate Cox regression analysis was used to screen for independent prognostic factors, resulting in 6-ORLs (AL035446.1, CRNDE, AC107294.1, SNAI3-AS1, AC083864.2, LINC02600), and patients were randomized into training cohort (332 samples) and validation cohort (329 samples), and risk scores (RS) were calculated for 6-ORLs in the training cohort by using multivariate Cox regression analysis to create predictive features. Among them, AL035446.1 and CRNDE were risk factors, while AC107294.1, SNAI3-AS1, AC083864.2 and LINC02600 were protective factors. The risk score was derived as follows: risk score = (0.139 × AL035446.1 expression) + (0.488 × CRNDE expression) + (− 0.483 × AC107294.1 expression) + (− 0.572 × SNAI3-AS1 expression) + (− 0.433 × AC083864.2 expression) + (− 0.467 × LINC02600 expression) (Supplementary Table S2). In the Pearson correlation analysis between OR-DEGs and ORLs, a total of 83 OR-DEGs were correlated with 6-ORLs. We selected the mRNAs among them with oxidative stress relevance score > 10 to demonstrate their association with 6-ORLs and RS (Fig. 2D). Subsequently, the results of Pearson correlation analysis of OR-DEGs and 6-ORLs were visualized by Cytoscape software to complete the co-expression network of OR-DEGs and 6-ORLs (Fig. 2E).

**Table 1:** Patient baseline characteristics of TCGA and CGGA glioma cohorts.

Characteristic	CGGA_325, N = 286^a^	CGGA_693, N = 429^a^	TCGA-test, N = 329^a^	TCGA-train, N = 332^a^	P value^b^
Grade					< 0.001
WHO II	86 (30%)	100 (23%)	116 (38%)	99 (33%)	
WHO III	68 (24%)	164 (38%)	119 (39%)	120 (40%)	
WHO IV	132 (46%)	165 (38%)	72 (23%)	78 (26%)	
Missing	0	0	22	35	
Gender					0.413
Female	110 (38%)	191 (45%)	125 (41%)	127 (43%)	
Male	176 (62%)	238 (55%)	182 (59%)	170 (57%)	
Missing	0	0	22	35	
Patient age					0.780
> 40	168 (59%)	249 (58%)	187 (61%)	182 (61%)	
≤ 40	118 (41%)	180 (42%)	120 (39%)	115 (39%)	
Missing	0	0	22	35	
IDH_mutation					< 0.001
Mutant	150 (52%)	236 (55%)	207 (63%)	216 (66%)	
Wildtype	136 (48%)	193 (45%)	119 (37%)	112 (34%)	
Missing	0	0	3	4	
1p19q codeletion					0.166
Codel	57 (20%)	90 (21%)	87 (26%)	80 (24%)	
Non-codel	229 (80%)	339 (79%)	242 (74%)	248 (76%)	
Missing	0	0	0	4	
MGMT methylation					< 0.001
Methylated	147 (51%)	251 (59%)	234 (74%)	240 (76%)	
Un-methylated	139 (49%)	178 (41%)	81 (26%)	76 (24%)	
Missing	0	0	14	16	

^a^n (%).

^b^Pearson's Chi-squared test.

### Validation of the 6-ORLs prognostic signature

**Figure 3:**
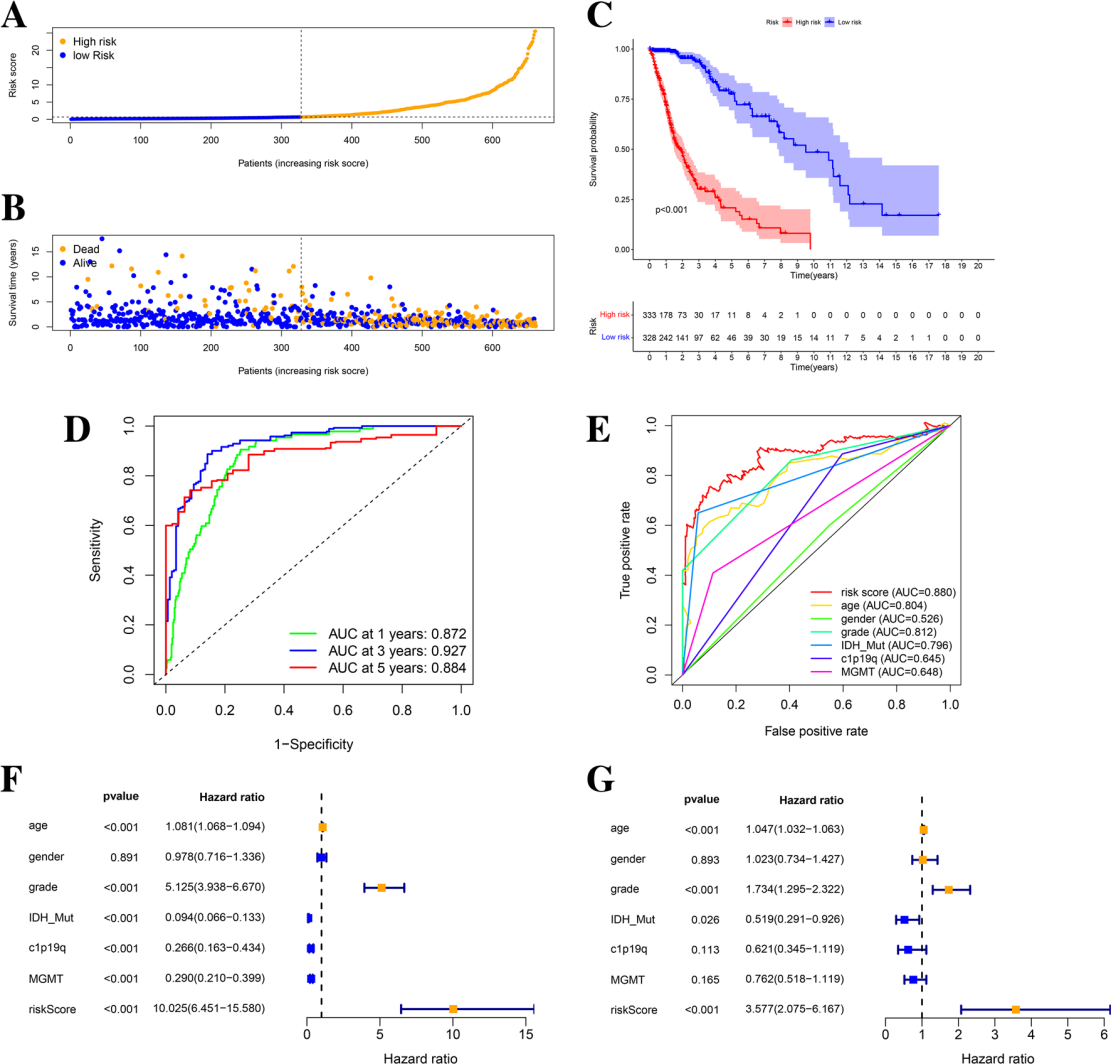
Validation of 6-ORLs prognostic signature in TCGA cohort. (**A**) Distribution of risk scores. (**B**) Survival status of patients with different risk scores. (**C**) Kaplan–Meier survival curve for different risk subgroups. (**D**) Time-dependent ROC curves and (**E**) Clinicopathological variables ROC curves for 6-ORLs prognostic signature. (**F**–**G**) Hazard ratio distributions of risk scores and clinicopathological variables in (**F**) univariate and (**G**) multivariate Cox regressions.

**Figure 4:**
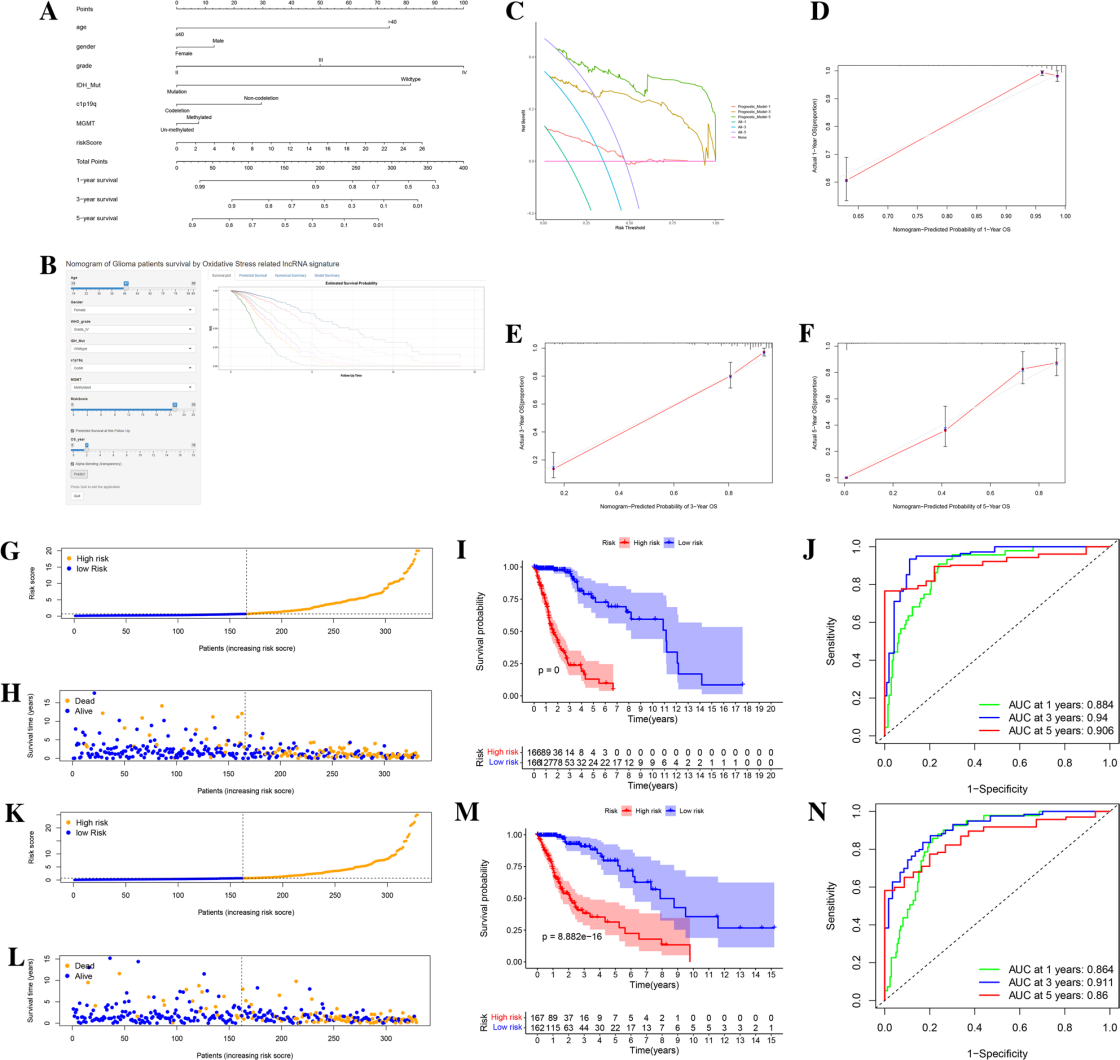
Nomogram and internal validation of TCGA cohorts for 6-ORLs prognostic signature. (**A**) Nomogram of risk scores and clinicopathological variables. (**B**) Online version of Nomogram (https://dnszy.shinyapps.io/Glioma_OS_lncRNA/). (**C**) Decision curve analysis (DCA) of Nomogram. (**D**–**F**) Calibration curves of Nomogram for 1, 3 and 5 years. (**G**–**N**) The risk score, patient survival status distribution, and Kaplan–Meier survival curve as well as ROC curves of 6-ORLs prognostic signature in the TCGA training cohort (**G**–**J**) and validation cohort (**K**–**N**).

To demonstrate the predictive capacity of the 6-ORLs prognostic signature for overall survival (OS) of patients in the TCGA dataset, we show the K–M curves with corresponding ROC curves for the prognostic signature in the whole TCGA glioma samples, the training cohort and the validation cohort, respectively. The demographic characteristics of the patients in both cohorts are shown in Table 1. in the whole TCGA glioma samples (Fig. 3A,B), the training cohort (Fig. 4G,H) and the validation cohort (Fig. 4K,L), the proportion of patients who died gradually rose with increasing risk scores. In the whole TCGA glioma samples, training cohort and validation cohort, K–M curves showed that RS significantly correlated with patients' OS, and patients in the high-risk group had considerably lower OS than those in the low-risk group, all of which were statistically significant (Figs. 3C, 4I,M, P < 0.001). For survival analysis of all glioma samples in TCGA, the ROC curve for RS showed a high area under the curve (AUC), with the best predictive power at year 3 (Fig. 3D, AUC at 3 years = 0.927). Combining RS with age, WHO grade, IDH mutation, short arm of chromosome 1 and long arm of chromosome 19 codeletion (1p19q codeletion), O6-methylguanine-DNA methyltransferase (MGMT) promoter methylation, and other clinicopathological variables were jointly included in the survival analysis, the ROC curve of RS had the best AUC (Fig. 3E, AUC of RS = 0.88). In both the training and validation cohorts, the ROC curve of RS still had a great AUC, suggesting that RS has excellent predictive efficacy for the prognosis of TCGA glioma patients (Fig. 4J, AUC at 3 years = 0.94; Fig. 4N, AUC at 3 years = 0.911). For univariate and multivariate Cox regression analysis of the whole TCGA glioma sample, RS showed the highest hazard ratio (HR) compared with other clinicopathological parameters, implicating RS as an independent prognostic factor for predicting OS in TCGA glioma patients (Fig. 3F, HR of risk score = 10.025; Fig. 3G, HR of risk score = 3.577).

### Relevance of 6-ORLs prognostic signature to clinicopathological parameters

To investigate whether 6-ORLs predictive signature still have predictive ability for OS in TCGA glioma patients in different clinicopathological variable classifications, TCGA glioma patients were classified according to age, gender, WHO grade, IDH mutation, 1p19q codeletion, MGMT promoter methylation into several groups. The results showed that RS still significantly predicted patients' OS in subgroups classified according to different clinicopathological variables (Fig. S2, P < 0.001), demonstrating that the predictive validity of RS was not affected by clinicopathological variables and had a robust predictive value for OS.

### Establishment and assessment of the RS-related nomogram

To intuitively predict the prognosis of glioma patients, we constructed a nomogram (Fig. 4A) using RS and clinicopathological variables (age, gender, WHO grade, IDH mutation, 1p19q codeletion, MGMT promoter methylation) and provided a web version (https://dnszy.shinyapps.io/Glioma_OS_lncRNA/) to facilitate the assessment of patient prognosis by clinicians (Fig. 4B). The internal validation and calibration of the nomogram was performed by 1000 bootstrap analyses. The results of the calibration curve showed that the actual survivability of patients and the predicted 1-, 3-, and 5-year survivability were in high agreement (Fig. 4D–F). The C-index of nomogram was 0.8556, demonstrating its excellent predictive efficacy. The decision curve analysis (DCA) of nomogram is shown in Fig. 4C. The risk threshold is the probability that a nomogram predicts whether a patient is at high risk, thus assisting clinicians in targeting treatment and intervention for high-risk patients. Net benefit value for different risk thresholds is given in the DCA. Decision curves showed that in nomogram predictions of risk probabilities for patients at 1, 3 and 5 years, if the risk threshold is between 20 and 50%, which is a more clinically reasonable threshold probability range (positive probability > 50%), aggressive antitumor therapy for these high-risk patients would result in more net benefit than any alternative strategy (considering all patients as high risk or considering all patients as low risk).

### Gene set enrichment analysis

To explore the molecular functional factors that resulted in significant differences in prognosis between patients in the high-risk and low-risk groups, we performed GSEA to identify the gene expression patterns of patients with different RS. Unlike functional enrichment analysis, GSEA analyzed the pathway enrichment of the sample expression profile by ranking all gene expression levels, and genes with insignificant expression differences (logFC < 1) were still included in the analysis, which helped to more accurately assess the gene expression patterns of the samples. We analyzed BP, CC, MF (Fig. S3A–C) in GO terms, KEGG pathway (Fig. S3D) and Reactome pathway (Fig. S3E) and selected the top 10 results of enrichment score (ES) for illustration. The results showed that, compared with the low-risk group, the high-risk group was significantly enriched in the biological functions of "cell activation", "collagen-containing extracellular matrix", and "carbohydrate binding" in GO terms. However, the "RNA processing" was significantly enriched in the low-risk group. In KEGG and Reactome, the high-risk group was significantly enriched in "ECM-receptor interaction", "Leukocyte transendothelial migration", "ECM proteoglycans", and "Hemostasis" pathways were significantly enriched. The results suggest that there are significant differences in the pathway activity and biological behavior of tumor cells between patients with different RS, which is probably one of the main factors causing the differences in prognosis.

### Comprehensive analysis of immune cell infiltration and immune function between high-risk and low-risk subgroups

**Figure 5:**
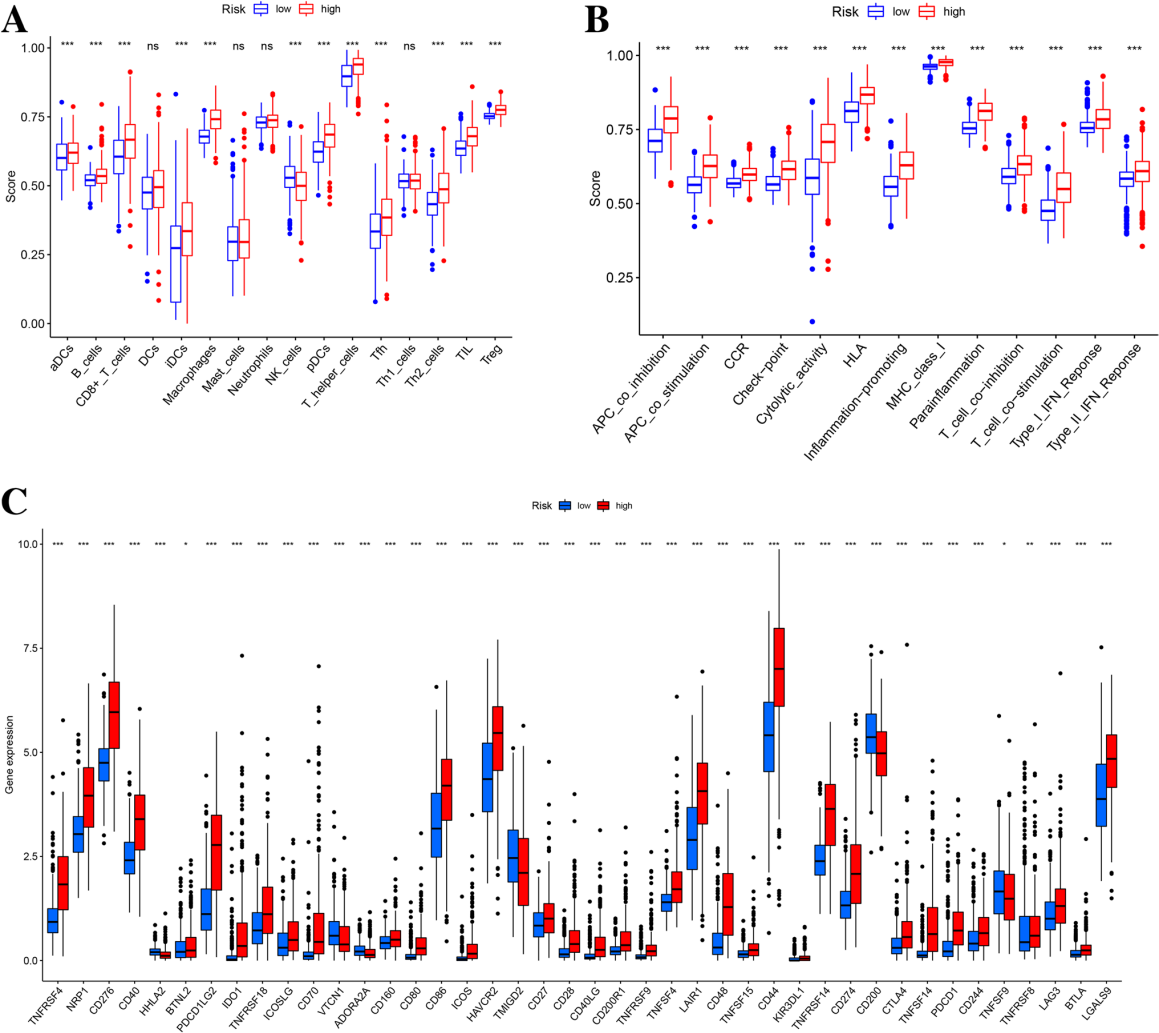
Differences in ssGSEA immune infiltration analysis and immune checkpoint-associated gene expression in high- and low-risk subgroups. (**A**) Immune cell infiltration analysis. (**B**) Immune-related functional analysis. (**C**) Differential expression of immune checkpoint-associated genes. *P < 0.05; **P < 0.01; ***P < 0.001; *ns* non-significant*.*

We used ssGSEA to estimate the correlation between RS and immune cells and immune function. Enrichment score of ssGSEA were evaluated using 16 immune cell subsets and 13 immune-related functional pathways, as shown in Fig. 5A,B. The results showed that the immune cells such as CD8+ T cells, interdigitating Dendritic Cells (iDCs), plasmacytoid dendritic cells (pDCs), macrophages and T helper cells were significantly different in the high and low risk groups (Fig. 5A). immune function scores such as APC co-inhibition, APC co-stimulation, Cytolytic activity, and inflammation promoting were higher in the high-risk group than in the low-risk group (Fig. 5B). This suggests that the tumor immune microenvironment of patients with high RS may have more active immune functions and greater immune response potential compared to patients with low RS. Subsequently, we performed a stratified analysis of the expression levels of 42 immune checkpoint-related genes, as shown in Fig. 5C. In the high-risk group, the expression levels of many immune checkpoint genes such as TNFRSF4, NRP1, CD276, PDCD1LG2, CD86, CD44, HAVCR2 were significantly up-regulated (P < 0.001) compared with the low-risk group, which may indicate that patients in the high-risk group have good responsiveness to some specific immune checkpoint inhibitors.

**Figure 6:**
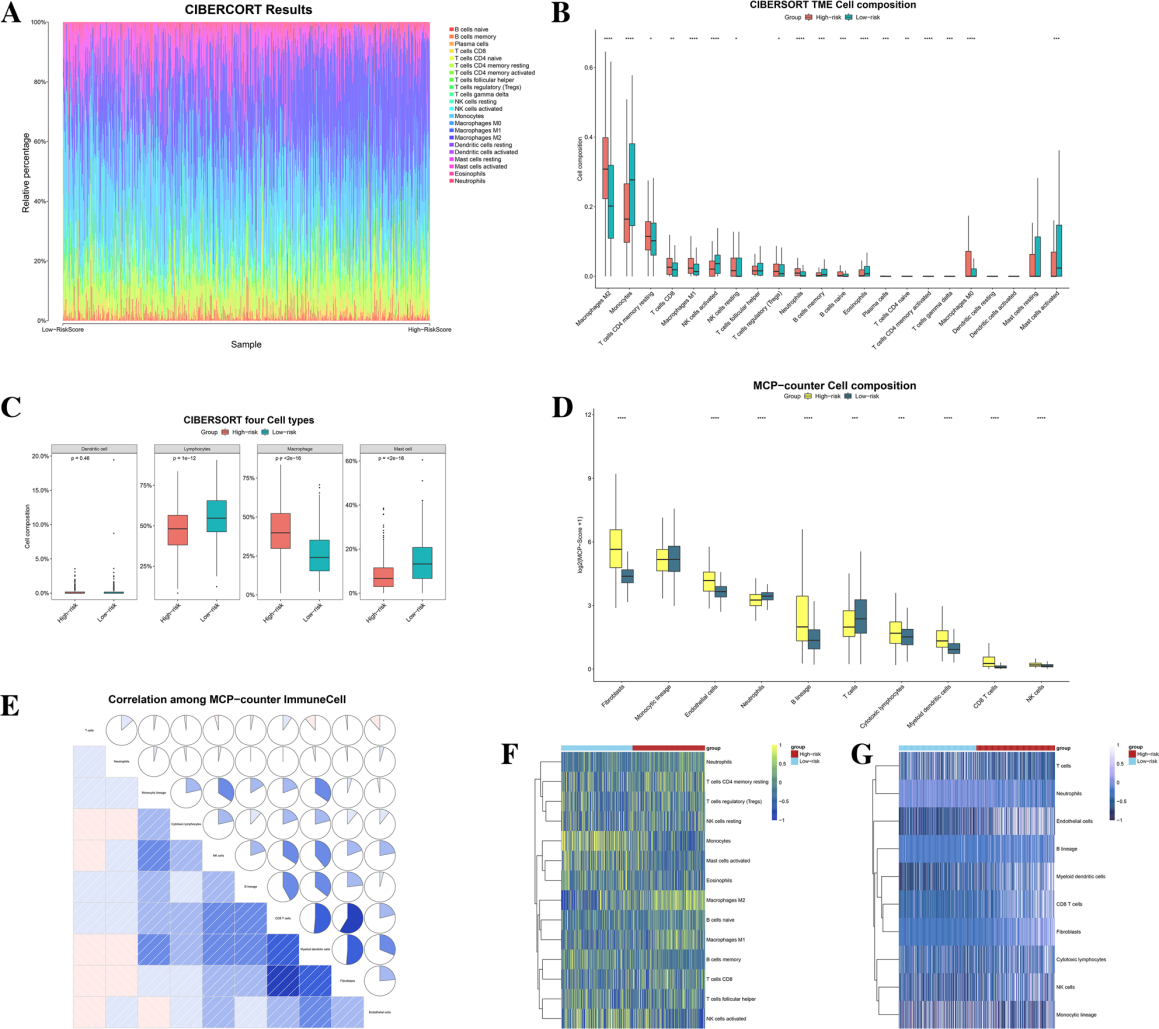
CIBERSORT and MCPcounter immune cell infiltration analysis in high- and low-risk subgroups. (**A**) Histogram of the proportion of CIBERSORT immune infiltrated cells. (**B**) Differences in the proportion of CIBERSORT immune infiltrating cells between high- and low-risk subgroups. (**C**) Proportion of different classes for immune cell infiltration in the high- and low-risk subgroups of CIBERSORT. (**D**) Proportion of MCPcounter immune cell infiltration in different risk subgroups. (**E**) Pearson correlation analysis between MCPcounter immune infiltrating cells. (**F**,**G**) Heatmap of CIBERSORT and MCPcounter immune infiltrate scores. *P < 0.05; **P < 0.01; ***P < 0.001; ****P < 0.0001.

**Figure 7:**
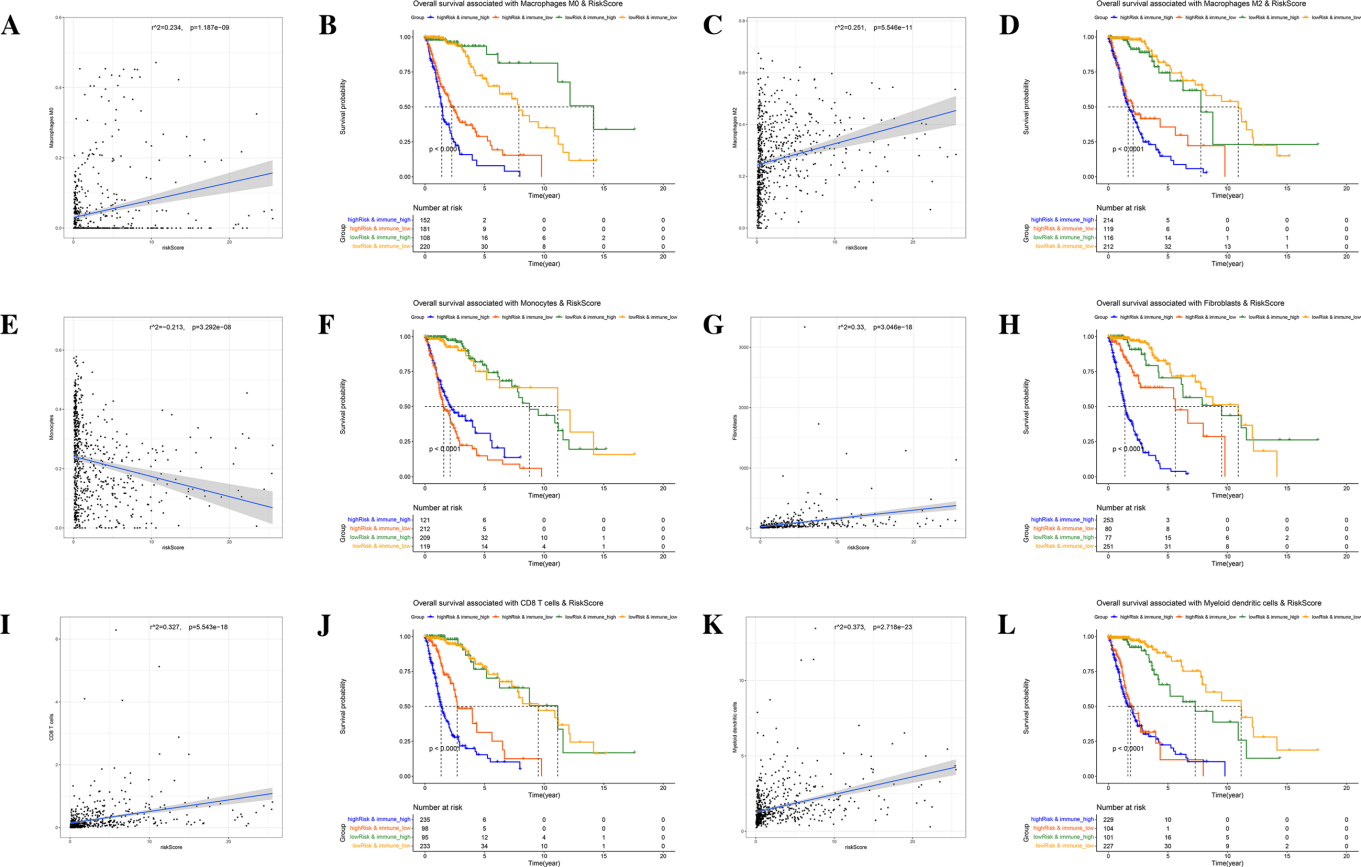
Pearson correlation analysis and survival analysis between immune score and risk score. (**A**–**F**) Pearson correlation analysis and Kaplan–Meier survival curve of macrophages M0 (**A**,**B**), macrophages M2 (**C**,**D**) and monocytes (**E**,**F**) in CIBERSORT. (**G**–**L**) Pearson correlation analysis and Kaplan–Meier survival curve of fibroblasts (**G**,**H**), CD8 T cells (**I**,**J**) and myeloid dendritic cells (**K**,**L**) in MCPcounter.

We also used CIBERSORT and MCPcounter to assess the difference in the abundance of immune cell infiltration in the high/low risk groups comprehensively (Figs. 6, 7). Figure 6A shows the histogram of the proportion of immune cell infiltration in CIBERSORT. Compared to the low-risk group, macrophage M2 infiltration was increased in the high-risk group, while monocytes infiltration was decreased (Fig. 6B, P < 0.001). The results of the CIBERSORT analysis were then summarized into 4 major immune cell subtypes, where macrophage infiltration was increased and lymphocytes and mast cell infiltration were decreased in the high-risk group (Fig. 6C, P < 0.001). MCPcounter results for the high/low risk groups showed that in the high-risk group, fibroblasts, endothelial cells, myeloid dendritic cells and B lineage infiltration were increased, while T cell infiltration was decreased (Fig. 6D, P < 0.001). Figure 6E demonstrates the Pearson correlation between immune infiltrating cells in MCPcounter results. Figure 6F,G shows heatmaps of the abundance of each immune infiltrating cell for CIBERSORT and MCPcounter, respectively.

We performed Pearson correlation analysis between the infiltration score of each immune cell in CIBERSORT in MCPcounter and patients' RS, performed K-M survival analysis to explore the association between the immune cell infiltration, RS and patients' prognosis. The results showed that macrophage M0, and macrophage M2 infiltration were positively correlated with RS, and monocytes infiltration was negatively correlated with RS in CIBERSORT (Fig. 7A,C,E). K–M curves showed that all three immune cell types could influence patient prognosis (P < 0.001). MCPcounter showed that infiltration of fibroblasts, CD8 T cells, and myeloid dendritic cells was positively correlated with RS (Fig. 7B,D,F). In the results of MCPcounter, fibroblasts, CD8 T cells, and myeloid dendritic cells infiltration were positively correlated with RS (Fig. 7G,I,K), and the K–M curves showed that myeloid dendritic cells had a greater prognostic impact on patients in the low-risk group, CD8 T cells and fibroblasts had a significant impact on the prognosis of patients in the high-risk group (P < 0.001), where fibroblasts infiltration could very significantly affect the survival of glioma patients with a potency that exceeded that of RS (Fig. 7H,J,L). Additional results are shown in Figs. S4 and S5.

In conclusion, the highly significant differences between the tumor immune microenvironment and the type of immune cell infiltration in patients in the high/low risk group, which correlated with RS and could have a significant impact on patient prognosis, demonstrated that the oxidative stress-related risk subgroups are probably with different tumor immune response patterns and may have a higher benefit when adopting different immunotherapy strategies during clinical treatment.

### Expression and prognostic differences of 6-ORLs in different grades of glioma

**Figure 8:**
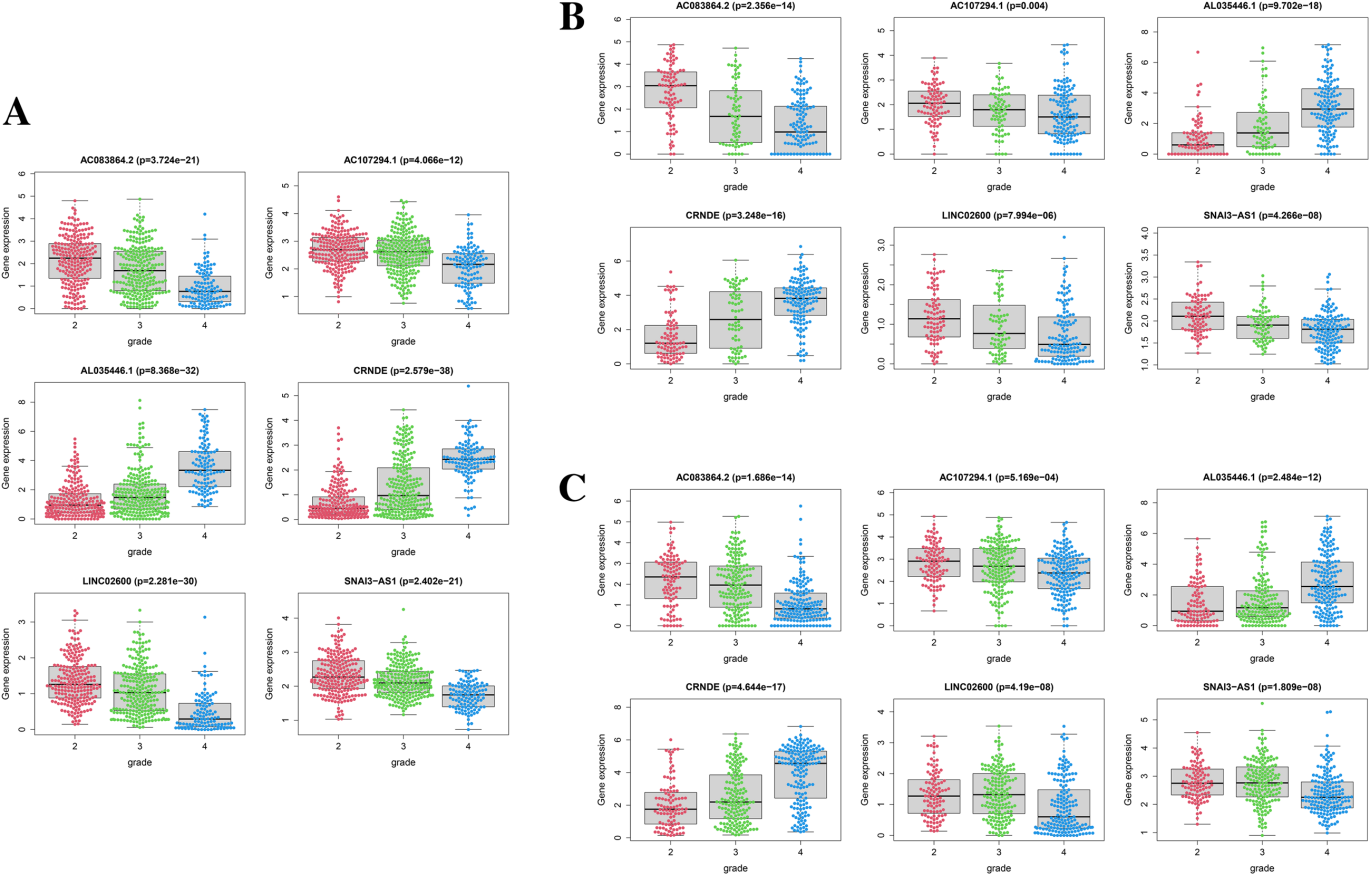
Expression levels of 6-ORLs in different WHO grades of glioma. (**A**) TCGA dataset. (**B**) CGGA-325 dataset. (**C**) CGGA-693 dataset.

**Figure 9:**
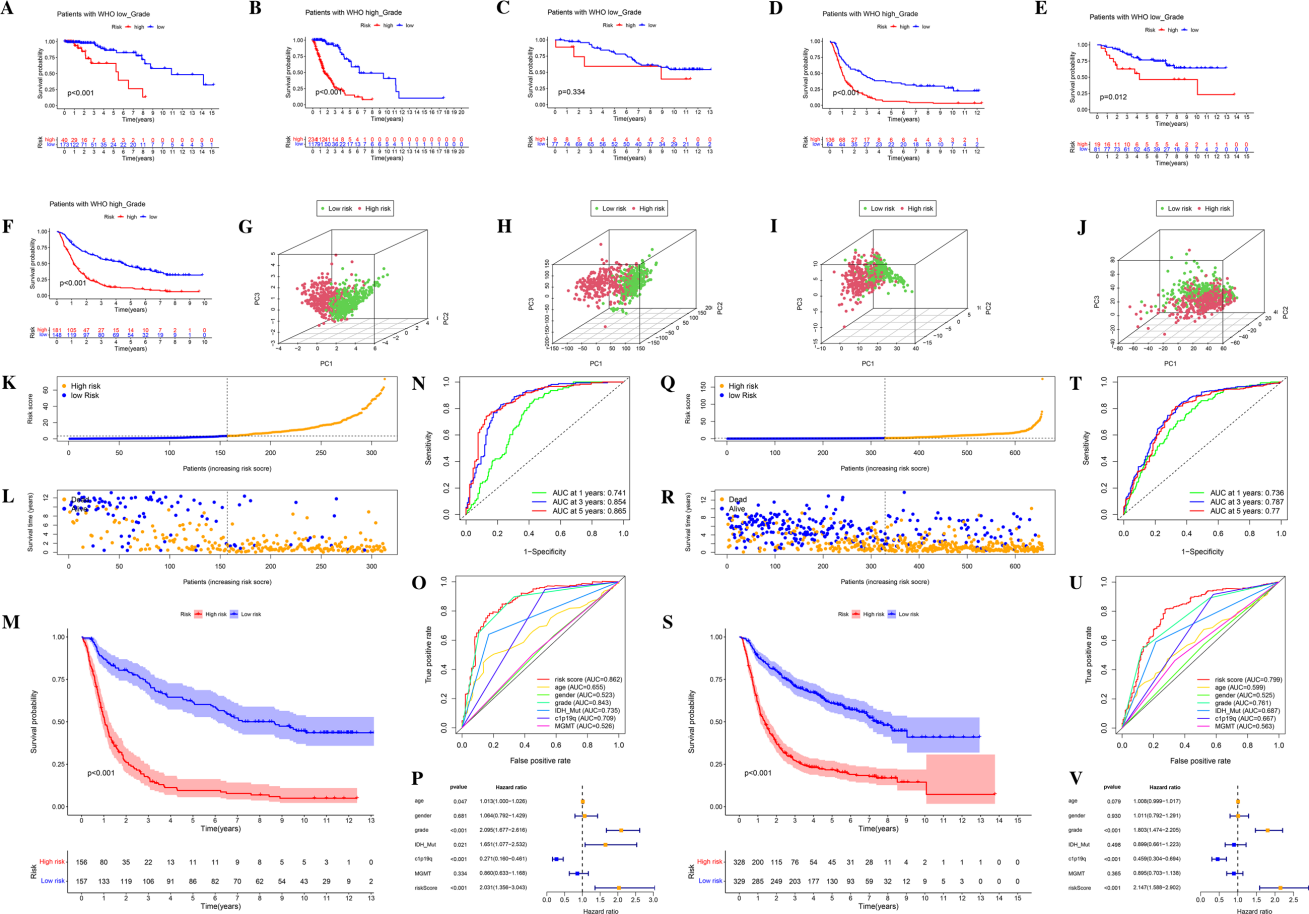
Kaplan–Meier curve of RS in LGG and HGG, principal component analysis and external validation in CGGA cohorts of 6-ORLs prognostic signature. (**A**–**F**) Kaplan–Meier curve of RS in LGG and HGG. (**A**,**B**) TCGA dataset. (**C**,**D**) CGGA-325 dataset. (**E**,**F**) CGGA-693 dataset. (**G**–**J**) Principal component analysis of different subgroups in RNA-seq expression profiles, OR-DEGs, all ORLs, and 6-ORLs expressions. (**K**–**V**) The risk score, patient survival status distribution, and Kaplan–Meier survival curve as well as ROC curves of 6-ORLs prognostic signature in the CGGA-325 cohort (**K**–**P**) and CGGA-693 cohort (**Q**–**V**).

Our study hopes to derive lncRNA prognostic signatures that can be adapted for patients with all grades of glioma through analysis of the combined dataset from different grades of glioma. However, considering the differences in gene expression, molecular typing and biological behavior of different grades of glioma, it is necessary to investigate whether 6-ORLs have differential expression and prognostic impact in high-grade and low-grade glioma. As shown in Fig. 8A, for the TCGA glioma dataset, 6-ORLs showed significant differences in expression among the WHO grades (P < 0.001). With increasing WHO grade, the expression of AL035446.1 and CRNDE was upregulated, while the expression of AC083864.2, AC107294.1, LINC02600, and SNAI3-AS1 was downregulated. We obtained similar results in the CGGA-325 and CGGA-693 datasets (Fig. 8B,C, P < 0.001). To further investigate the prognostic impact of RS in different grades of glioma, we distinguished all glioma patient samples into high-grade glioma (WHO-HGG) and low-grade glioma (WHO-LGG) according to WHO classification standards^1^. WHO-HGG included WHO grade III and IV, and WHO-LGG included WHO grade I and II. The results showed that the risk subgroups of 6-ORLs prognostic signature had significant prognostic differences (P < 0.01) in both WHO-HGG and WHO-LGG patients in the TCGA (Fig. 9A,B) and CGGA-693 (Fig. 9E,F) datasets. In the CGGA-325 dataset, the risk subgroup had a significant prognostic difference in WHO-HGG (Fig. 9D, P < 0.01) but no significant prognostic difference in WHO-LGG (Fig. 9C, P = 0.334), which probably resulted from the smaller number of patients in the high-risk subgroup of WHO-LGG in this dataset. In conclusion, the expression of 6-ORLs was significantly differed in different grades of glioma, and the prognostic differences in risk subgroups were remarkable both in WHO-HGG and WHO-LGG.

### Principal component analysis

To verify whether the gene expression of RNA-seq expression profiles, OR-DEGs, all ORLs, and 6-ORLs could distinguish the high/low risk groups well, we plotted a three-dimensional scatter plot of PCA to demonstrate the distribution of gene expression in different dimensions in agreement with the risk subgroups. The results showed that the risk subgroups were well matched with the PCA results of RNA-seq expression profiles, OR-DEGs, all ORLs, and 6-ORLs (Fig. 9G–J).

### External validation of 6-ORLs prognostic signature

To validate the robustness of the 6-ORLs prognostic signature and further demonstrate its predictive ability for the prognosis of glioma patients, we used the CGGA-325 and CGGA-693 datasets as external validation (test cohorts) for this study. Baseline characteristics of the patients are shown in Table 1. First, 6-ORLs prognostic signature was constructed in CGGA-325 and CGGA-693 datasets using multivariate Cox regression analysis, and high/low risk groups were identified in the same way. Subsequently, K-M survival analysis and ROC curve validation were performed, and HR levels of multivariate Cox regression were demonstrated, as shown in Fig. 9K–V. The results of our analysis showed that 6-ORLs still had excellent predictive efficacy for prognosis of glioma patients in the CGGA-325 and CGGA-693 datasets, with significant prognostic differences in the high/low risk groups (Fig. 9M,S, P < 0.001). The ROC curves showed that all CGGA test cohorts of RS had excellent 1-year, 3-year and 5-year AUC and were higher than other clinicopathological variables, further demonstrating the validity of 6-ORLs in predicting prognosis (Fig. 9N,O,T,U). In test cohorts, RS were available as independent prognostic factors with absolute values of HR greater than other clinicopathological variables and were significant risk factors (Fig. 9P,V).

In addition, we likewise performed stratified survival analysis of clinicopathological variables, ssGSEA immune cell and immune function analysis, CIBERSORT and MCPcounter immune infiltration analysis, correlation between immune infiltration abundance and RS, survival analysis of immune scores, immune checkpoint-associated gene expression analysis, and differential expression analysis of 6-ORLs in clinicopathological variables for CGGA test cohorts. In addition, we likewise performed stratified survival analysis of clinicopathological variables, ssGSEA immune cell and immune function analysis, CIBERSORT and MCPcounter immune infiltration analysis, correlation between immune infiltration abundance and RS, survival analysis of immune scores and immune checkpoint-associated gene expression analysis of 6-ORLs in clinicopathological variables for CGGA test cohorts (Figs. S6–S9). Although the correlation between the abundance of some immune cell infiltrates and RS showed an inconsistent intensity with the TCGA dataset, such as macrophage M2 and CD8 T cells (Figs. S8B,F, S9A,D), this difference may be indicative of variations of gene expression patterns in glioma patients from different countries and regions, almost all the above analyses showed a trend consistent with the results of the TCGA dataset analysis.

The results of the above analysis demonstrate that, the different risk subgroups identified by the 6-ORLs prognostic signature in gliomas can stably distinguish prognostic differences in patients, stratified characteristics of clinicopathological variables, tumor microenvironment landscapes, immune cell infiltration patterns and expression levels of immune checkpoint-related genes.

### Differential expression of 6-ORLs and validation by RT-qPCR

**Figure 10:**
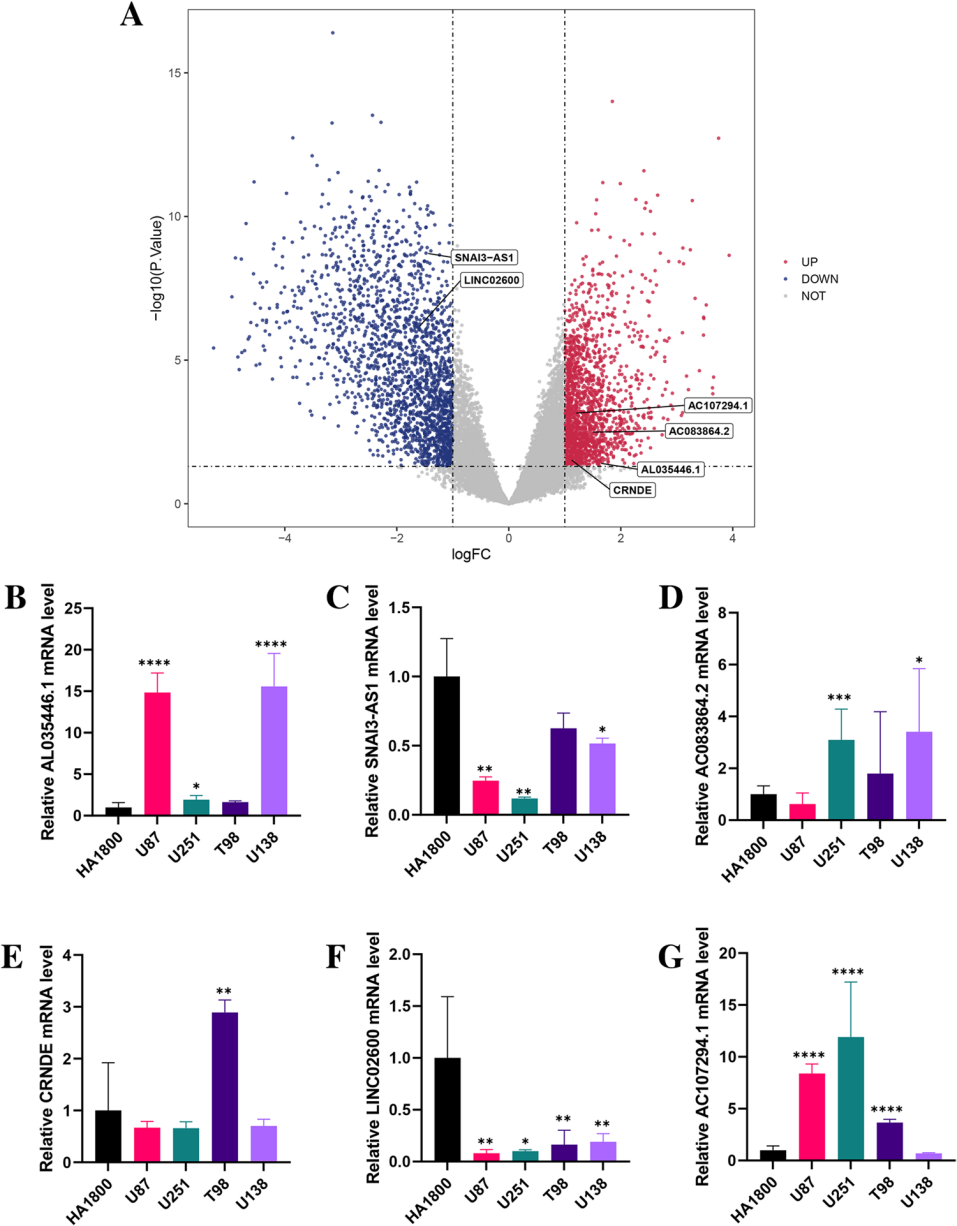
Differential expression of 6-ORLs in TCGA dataset and validation by RT-qPCR. (**A**) Volcano plot of Limma differential expression analysis of tumor samples and paracancer samples in TCGA glioma dataset. Locations of 6-ORLs are labeled in the figure. (**B**–**G**) Expression levels of 6-ORLs in astrocyte line HA1800 and glioma cell lines U87, U251, T98 and U138. Welch t-test was performed for the significance level of the difference in expression of 6-ORLs in glioma cell lines and HA1800. *P < 0.05; **P < 0.01; ***P < 0.001; ****P < 0.0001.

**Figure 11:**
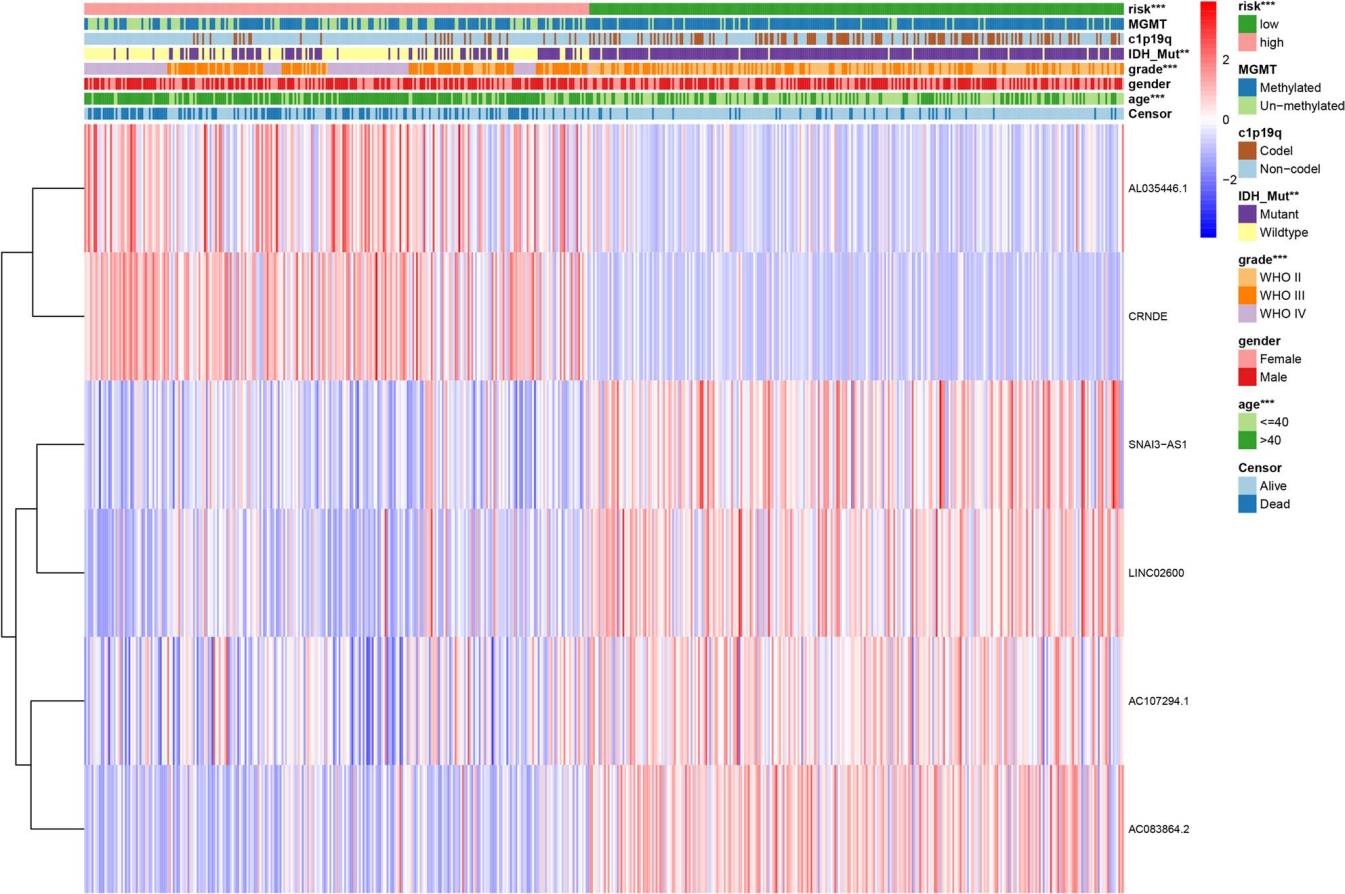
Heatmap of 6-ORLs gene expression in association with clinicopathological variables and risk subgroups. *P < 0.05; **P < 0.01; ***P < 0.001*.*

To explore the difference in expression levels of 6-ORLs in glioma and paraneoplastic tissues, we performed Limma differential expression analysis on cancer samples and normal samples from the TCGA glioma dataset, as shown in Fig. 10A. Notably, although our study confirmed that AC107294.1 and AC083864.2 were down-regulated in the high-risk group (Fig. 11), their expression levels in glioma tissue were up-regulated compared to the paraneoplastic tissue. To further validate the results of the analysis, we performed RT-qPCR to verify the expression levels of 6-ORLs in the astrocyte line HA1800 and glioma cell lines U87, U251, T98, and U138 (Fig. 10B–G). The results showed that compared to HA1800 cell line, AL035446.1 expression was upregulated in U87, U251 and U138 cell lines, CRNDE was upregulated in T98 cell line, AC083864.2 was upregulated in U251 and U138 cell lines, AC107294.1 was upregulated in U87, U251, and T98 cell lines, SNAI3-AS1 was downregulated in U87, U251 and U138 cell lines, and LINC02600 was downregulated in U87, U251, T98 and U138 cell lines. This result confirms our analysis of the TCGA dataset. A part of cell lines did not present differential expression of 6-ORLs genes, which may be due to the fact that 6-ORLs are mainly involved in oxidative stress-related functions in different glioma subtypes rather than functional differences between glioma and paraneoplastic tissues.

## Discussion

In this study, we developed a prognostic signature consisting of 6-ORLs to assess oxidative stress characteristics associated with clinical prognosis in gliomas. Based on the results of the enrichment analysis, it is reasonable to assume that 6-ORLs can distinguish oxidative stress status in different glioma patients. The prognostic models were screened based on Pearson correlation coefficients of ORGs with lncRNAs and LASSO dimension reduction screening of ORLs. The predictive ability of the models for prognosis was superior in both internal validation of the TCGA dataset and external validation of the CGGA dataset. Multivariate Cox regression analysis further confirmed that risk scores can be used as independent prognostic factors. A nomogram containing risk scores for 6-ORLs and several clinicopathological variables further quantified the predictive efficacy of the prediction model for prognosis of glioma patients. Stratified analysis for clinicopathological variables showed that the 6-ORLs risk subgroup had prognostic differences across clinicopathological variables, largely excluding bias in the results due to other confounders. For the different risk subgroups, we analyzed their different biological functions and immune infiltration status, confirming the robust classification of risk scores for differences in biological behavior and treatment response of tumors in glioma patients. 6-ORLs were significantly differentially expressed in different grades of glioma, and subsequent stratified survival analysis showed statistically prognostic differences in both HGG and LGG for our prognostic signature. Finally, we evaluated the differential expression of 6-ORLs in glioma and paraneoplastic tissues using the TCGA dataset and validated at the cell line level by RT-qPCR.

Glioma is the most common primary brain tumor in adults and usually has a poor prognosis^38^. The median survival of glioblastoma has been reported to be only 14.6 months, and the 5-year survival rate of diagnosed patients is less than 5%^39^. In recent years, the biological mechanisms regarding oxidative stress and its effects on tumor cells have been intensively explored. Reactive oxygen species (ROS) levels are significantly higher in tumor cells than in normal cells, and high ROS levels are closely associated with biological behaviors such as proliferation, invasion and metastasis of tumor cells^40^. Further more, oxidative stress triggered by reactive oxygen species, or reactive nitrogen species (RNS) can lead to cellular DNA damage, inhibit cellular reactive enzymes, affect apoptosis and proliferation, which potentially increases the incidence of glioma^41,42^. It has been found that peroxiredoxin 4 (PRDX4) is upregulated in glioma stem cells (GSC) in the central region of the tumor, resulting in low levels of ROS production in GSC even in a hypoxic environment^43^. Oxidative stress is also of considerable interest in the treatment of glioma^44^. Studies have shown that oxidative stress influences temozolomide resistance^43^ and radiation therapy resistance^45^ in glioma. Therefore, there is an urgent need to find a molecular marker that conveniently assesses the level of oxidative stress in glioma for diagnosing the prognosis of glioma patients and developing individualized treatment strategies.

Colorectal Neoplasia Differentially Expressed (CRNDE) is an lncRNA located at the atypical locus hCG_1815491 on chromosome 16, adjacent to the IRX5 gene, sharing the same bidirectional promoter, and was first named for its upregulated expression in colorectal cancer (CRC)^46,47^.CRNDE has been reported to be highly expressed in a variety of cancers, such as hepatocellular carcinoma, lung cancer, renal cell carcinoma, and glioma^48^. CRNDE can be involved in Wnt/β-catenin^49,50^, PI3K/AKT/mTOR^51,52^, Ras/mitogen-activated protein kinase (MAPK)^53^ and Notch1^54^, and several other cancer-related signaling pathways to promote tumor cell proliferation, inhibit apoptosis, promote invasion, migration and treatment resistance, which are considered as oncogenes^55,56^. It has been demonstrated that knockdown of CRNDE in human glioma cell lines U87 and U251 enhances the sensitivity of temozolomide chemotherapy by regulating autophagy-related functions^57^. Snail Family Transcriptional Repressor 3 Antisense RNA 1 (SNAI3-AS1) is a lncRNA located in the amplified 16q24 motif^58^, and its specific biological function has not been clarified. In recent years, SNAI3-AS1 has been reported to promote the proliferation and metastasis of hepatocellular carcinoma possibly by regulating the PEG10, UPF1 and TGF-β signaling pathway^58,59^. In addition, SNAI3-AS1 was reported to be possibly associated with epithelial-mesenchymal transition in glioma^60^. Although other 6-ORLs genes has not been reported, the findings of our study suggest that they are likely to regulate important biological functions during oxidative stress in glioma.

In a functional enrichment analysis of mRNAs associated with 6-ORLs, we identified some significantly enriched pathways and biological behaviors. Hypothalamic hormone (GnRH) was first identified by Schally et al. in 1971^61^, and subsequent studies demonstrated its regulatory role on human growth and reproductive functions^62^. In recent years, the role of the GnRH receptor (GnRH-R) in hormone-dependent tumors (such as breast, prostate, and ovarian cancers) has been explored and widely used in antitumor therapy. Available studies have demonstrated that GnRH-R is expressed in a variety of tumor tissues, including non-reproductive mesenchymal tumors such as melanoma, glioblastoma, lung cancer and pancreatic cancer^63^. Application of GnRH agonists to activate GnRH-R may lead to significant antitumor effects such as proliferation, anti-metastasis, and anti-angiogenesis^64^, which may be related to intracellular signaling pathways coupled to GnRH-R in different tissues^65^. Transforming growth factor-β (TGF-β) is a family of structurally related proteins that are closely associated with a variety of cellular functions, including proliferation, apoptosis, differentiation, epithelial-mesenchymal transition (EMT) and migration, regulate protein serine/threonine kinase activity and lead to phosphorylation of the intracellular effector SMAD protein, which promote tumor invasion and metastasis^66^. Activation of the TGF-β pathway has been shown to be associated with aggressiveness, tumor cell stemness and worse prognosis in glioma^67^.

Significant differences in the tumor immune microenvironment were found in the different risk subgroups of the 6-ORLs prognostic signature. Infiltration of fibroblasts and macrophages M0 was upregulated in high-risk subgroups and was associated with poor prognosis in both TCGA and CGGA datasets. Cancer-associated fibroblasts (CAFs) are suggested to be associated with invasive niche in glioblastoma and are mediated via TGF-β^68^. CAFs may influence glioma development through multiple pathways, leading to worse prognosis. It has been reported that αv integrins can affect the recognition of glioblastoma stem cells by natural killer cells (NK cells) and recruit CAFs to tumor cells by regulating TGF-β-related pathways^67,69^. Recent studies have indicated that CAFs have the potential to lead to enhanced drug resistance in glioblastoma by affecting its blood–brain barrier (BBB) function^70^. Tumor-associated macrophages (TAMs) infiltrate abundantly in glioma and commonly correlate with poor prognosis^71^. There is evidence that the level of TAM infiltration is associated with recurrence of GBM^72^. In an immune infiltration analysis of glioma single cell sequencing data from TCGA, CGGA databases, it was found that the abundance of macrophage M0 correlated with glioma mesenchymal phenotype and IDH wild type, with worse prognosis in patients^73^. Glutaredoxin, a redox regulator, and its encoding gene GLRX were shown to be specifically expressed in macrophage M0, interferes with immune checkpoint function and inflammatory response in glioma, corresponding to worse survival in patients^74^. Our study suggests that oxidative stress may induce an immune microenvironment with increased infiltration abundance of TAMs with CAFs, which could shed some light on subsequent studies.

There are still limitations to our study. Firstly, the prognostic signature was established using only public databases, and only used retrospective data for survival analysis. Secondly, there is a lack of clinical samples for validation. Finally, only the validation of the expression level of 6-ORLs in cell lines was completed, without further exploring the target genes and molecular functions.

## Conclusion

We identified oxidative stress-related lncRNAs in glioma patients and screened for the critical genes to establish a prognostic signature. The predictive power of survival and immune cell infiltration were assessed for different risk subgroups and independently validated by external public datasets. The results showed that the prognostic signature is a predictor of survival and treatment response in glioma patients, with significant differences in tumor-related molecular characteristics and biological behavior in different subgroups. This may provide a new meaningful orientation for individualized treatment strategies and survival assessment of glioma patients.

## Supplementary Information

Supplementary Information 1.

Supplementary Information 2.

Supplementary Information 3.

Supplementary Information 4.

Supplementary Information 5.

Supplementary Information 6.

Supplementary Information 7.

Supplementary Information 8.

Supplementary Information 9.

Supplementary Information 10.

Supplementary Information 11.

Supplementary Information 12.

## Data Availability

The datasets generated and analyzed during the current study are available from public databases, TCGA (http://cancergenome.nih.gov/abouttcga), CGGA (http://www.cgga.org.cn/about.jsp), GENCODE (https://www.gencodegenes.org/pages/gencode.html) and GeneCards (https://www.genecards.org/Guide/AboutGeneCards) databases.
