# Supplementary figures and images for: Comprehensive analysis of oxidative stress-related lncRNA signatures in glioma reveals the discrepancy of prognostic and immune infiltration
Source: Sci Rep. 2023 May 12;13:7731. doi: 10.1038/s41598-023-34909-y (PMC10182081; doi:10.1038/s41598-023-34909-y)

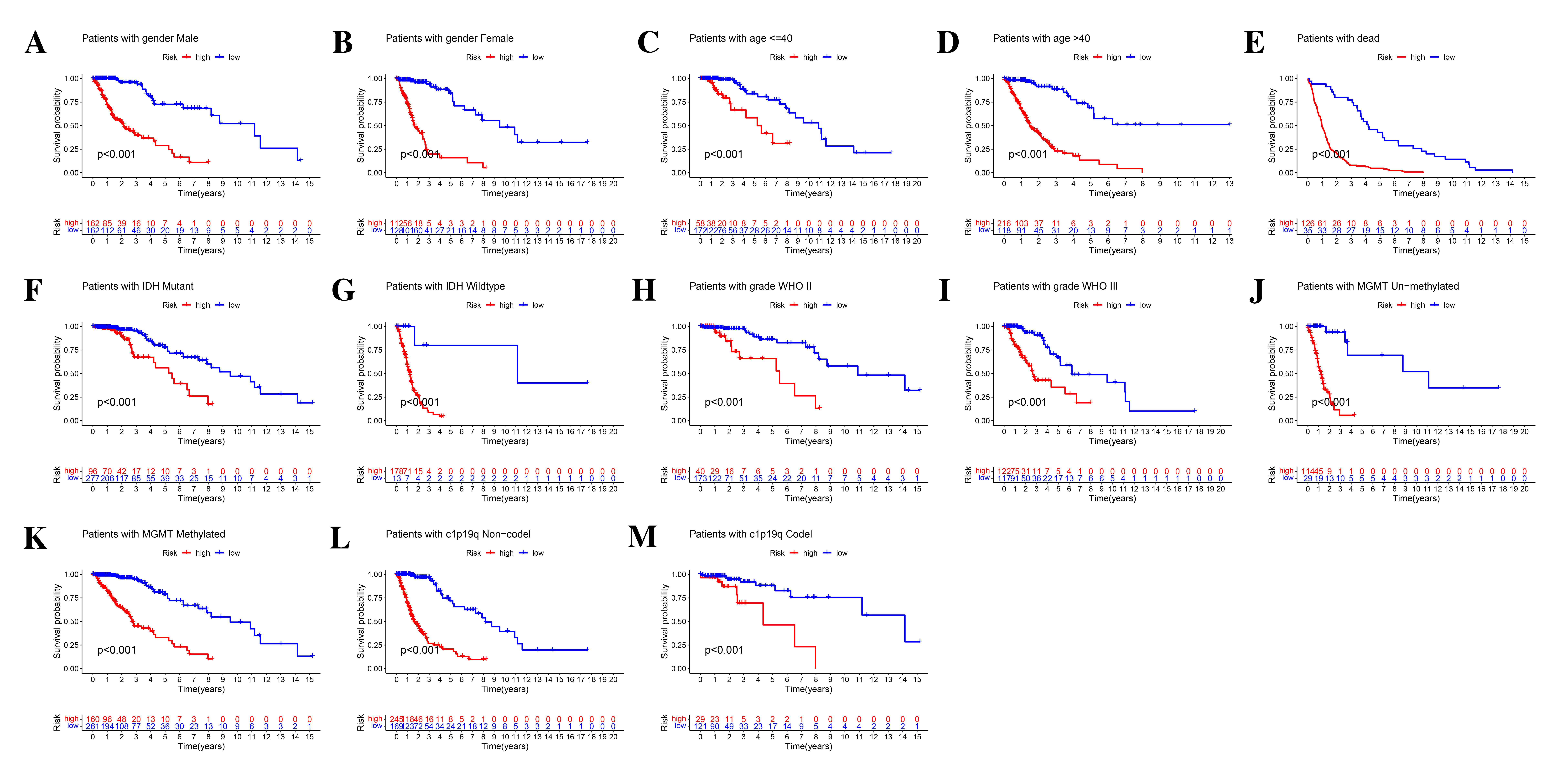

Supplement: Supplementary file 2 — Supplementary Information 2. [file 41598_2023_34909_MOESM2_ESM.tif]

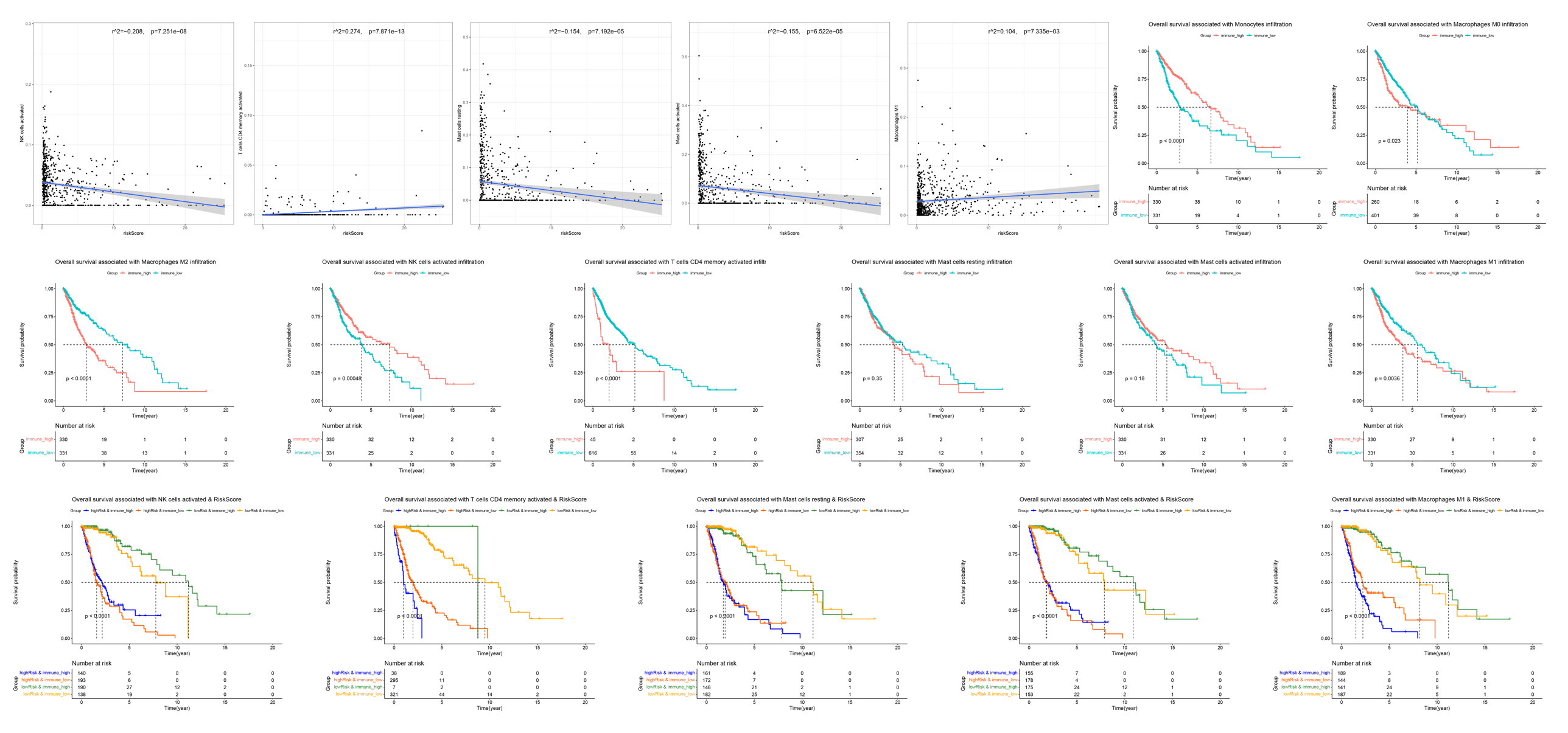

Supplement: Supplementary file 4 — Supplementary Information 4. [file 41598_2023_34909_MOESM4_ESM.tif]

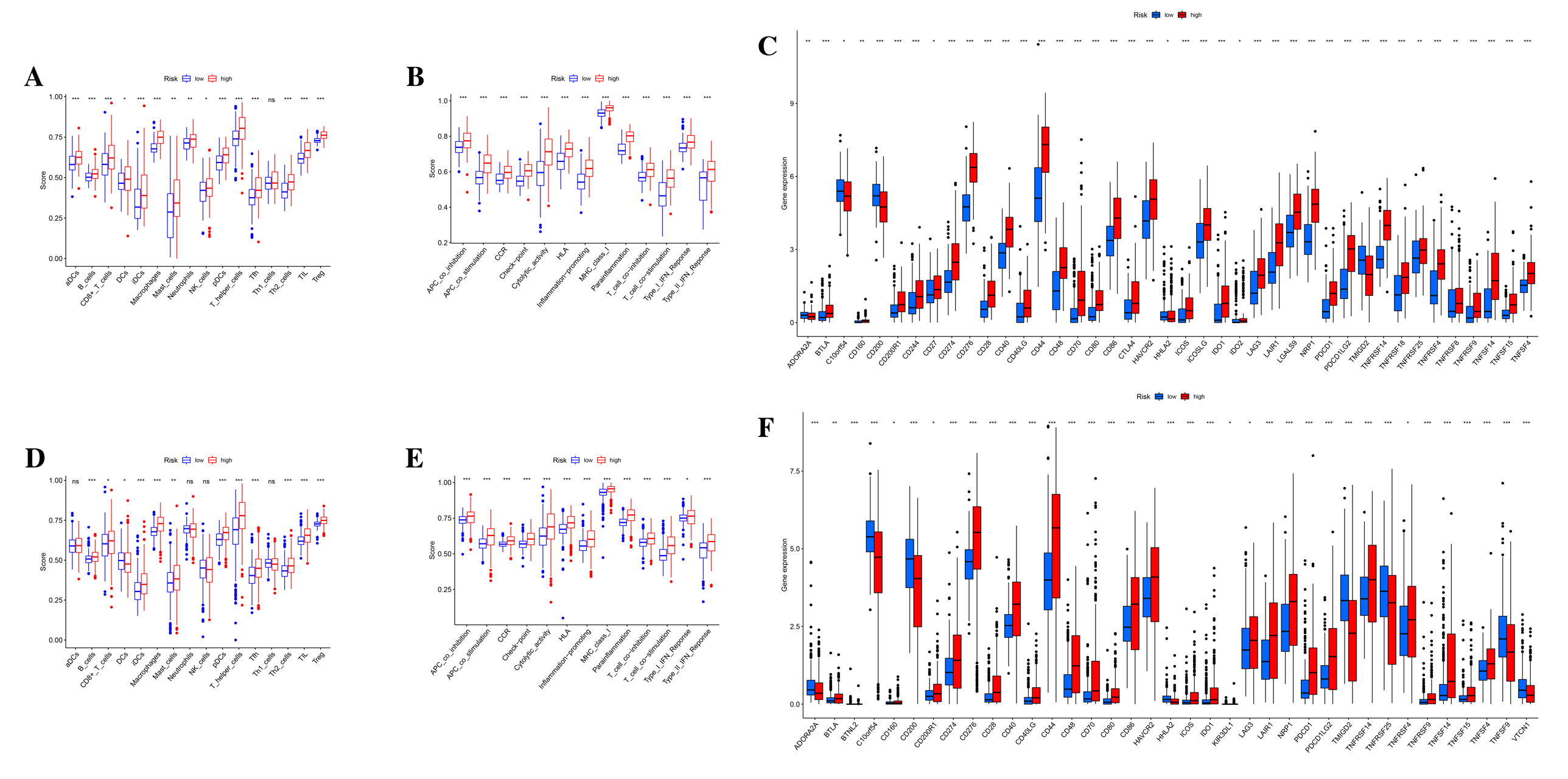

Supplement: Supplementary file 7 — Supplementary Information 7. [file 41598_2023_34909_MOESM7_ESM.tif]
