# Supplementary material for: Comprehensive analysis of oxidative stress-related lncRNA signatures in glioma reveals the discrepancy of prognostic and immune infiltration
Source: Sci Rep. 2023 May 12;13:7731. doi: 10.1038/s41598-023-34909-y (PMC10182081; doi:10.1038/s41598-023-34909-y)
Supplement: Supplementary file 10 — Supplementary Information 10. [file 41598_2023_34909_MOESM10_ESM.docx]

FIGURE S1 | Functional enrichment analysis of OR-DEGs. (A) GO analysis of necroptosis-related OR-DEGs. (B) KEGG analysis of OR-DEGs. BP, biological process; CC, cellular components; MF, molecular function.

FIGURE S2 | (A-M) Stratified survival analysis of 6-ORLs prognostic signature in a TCGA cohort with different clinicopathological variables.

FIGURE S3 | Gene Set Enrichment Analysis (GSEA) of high and low risk subgroups. (A-C) GSEA analysis of biological process (BP), cellular component (CC), and molecular function (MF) in GO terms. (D-E) GSEA analysis of KEGG, Reactome pathway.

FIGURE S4 | Pearson correlation analysis and survival analysis of partial CIBERSORT immune infiltration scores with RS in the TCGA cohort.

FIGURE S5 | Pearson correlation analysis and survival analysis of partial MCPcounter immune infiltration scores with RS in the TCGA cohort.

FIGURE S6 | (A-B) Stratified survival analysis of different clinicopathological variables in high- and low-risk subgroups of the CGGA-325 (A) and CGGA-693 (B) cohorts with 6-ORLs prognostic signature.

FIGURE S7 | (A-F) ssGSEA immune cell infiltration, immune function and immune checkpoint-related gene expression levels in the CGGA-325 (A-C) and CGGA-693 (D-F) cohorts.

FIGURE S8 | (A-H) Pearson correlation and survival analysis of CIBERSORT immune infiltration proportions, immune scores and risk scores in high and low risk subgroups in CGGA-325 (A-D) and CGGA-693 (E-H). Demonstrate selected results with statistically significant differences.

FIGURE S9 | (A-F) Pearson correlation and survival analysis of MCPcounter immune scores and risk scores in high and low risk subgroups in CGGA-325 (A-C) and CGGA-693 (D-F). Demonstrate selected results with statistically significant differences.

TABLE S1 | Pearson correlation analysis of OR-DEGs and lncRNAs.

TABLE S2 | Multivariate COX regression analysis of 6-ORLs and risk coefficients of prognostic signature.
